# Assessment of the practical impact of adjusting beta-lactam dosages based on therapeutic drug monitoring in critically ill adult patients: a systematic review and meta-analysis of randomized clinical trials and observational studies

**DOI:** 10.1038/s41598-024-58200-w

**Published:** 2024-04-02

**Authors:** Eszter Gulyás, István László Horváth, Marie Anne Engh, Stefania Bunduc, Fanni Dembrovszky, Péter Fehérvári, András Bánvölgyi, Dezső Csupor, Péter Hegyi, Gellért Balázs Karvaly

**Affiliations:** 1 Centre for Translational Medicine, Semmelweis University, Budapest, Hungary; 2 University Pharmacy Department of Pharmacy Administration, Semmelweis University, Budapest, Hungary; 3 Department of Laboratory Medicine, Semmelweis University, 4 Nagyvarad ter, Budapest, 1089 Hungary; 4 Institute for Translational Medicine, Medical School, University of Pécs, Pécs, Hungary; 5 Carol Davila University of Medicine and Pharmacy, Bucharest, Romania; 6 Fundeni Clinical Institute, Bucharest, Romania; 7 First Department of Medicine, University of Pécs, Pécs, Hungary; 8 János Szentágothai Research Center, University of Pécs, Pécs, Hungary; 9 Department of Biostatistics, University of Veterinary Medicine, Budapest, Hungary; 10 Department of Dermatology, Venereology and Dermatooncology, Semmelweis University, Budapest, Hungary; 11 Department of Clinical Pharmacy, University of Szeged, Szeged, Hungary; 12 Division of Pancreatic Diseases, Heart and Vascular Center, Semmelweis University, Budapest, Hungary

**Keywords:** Beta-lactam, Therapeutic drug monitoring, Meta-analysis, ICU, Critically ill, Antimicrobial therapy, Bacterial infection

## Abstract

An estimated 70% of critically ill patients receive antibiotics, most frequently beta-lactams. The pharmacokinetic properties of these substances in this patient population are poorly predictable. Therapeutic drug monitoring (TDM) is helpful in making personalized decisions in this field, but its overall impact as a clinical decision-supporting tool is debated. We aimed to evaluate the clinical implications of adjusting beta-lactam dosages based on TDM in the critically ill population by performing a systematic review and meta-analysis of available investigations. Randomized controlled trials and observational studies were retrieved by searching three major databases. The intervention group received TDM-guided beta-lactam treatment, that is, at least one dose reconsideration based on the result of the measurement of drug concentrations, while TDM-unadjusted dosing was employed in the comparison group. The outcomes were evaluated using forest plots with random-effects modeling and subgroup analysis. Eight eligible studies were identified, including 1044 patients in total. TDM-guided beta-lactam treatment was associated with improved clinical cure from infection [odds ratio (OR): 2.22 (95% confidence interval (CI): 1.78–2.76)] and microbiological eradication [OR: 1.72 (CI: 1.05–2.80)], as well as a lower probability of treatment failure [OR: 0.47 (CI: 0.36–0.62)], but the heterogeneity of studies was remarkably high, especially in terms of mortality (70%). The risk of bias was moderate. While the TDM-guided administration of beta-lactams to critically ill patients has a favorable impact, standardized study designs and larger sample sizes are required for developing evidence-based protocols in this field.

## Introduction

Therapeutic drug monitoring (TDM) has been defined as the core component of individualizing drug therapy by leading international professional bodies^1,2^. Nevertheless, in critical care, the role of TDM in improving the efficacy and safety of antibiotic treatments is under intense debate^3–7^. Among the intravenous medications employed, broad-spectrum beta-lactams (penicillins, cephalosporins, and carbapenems) have received particular attention due to their extensive and frequently empirical use and the limited evidence available on their optimal administration. Although toxicity is less of a concern than with glycopeptide or aminoglycoside antimicrobials, underdosing is a threat to patients due to the existence of various dosing regimens, the substantial interindividual variability in the pharmacokinetic properties of these substances, and the instability of the patients’ clinical status^7,8^. The number of position papers and expert opinions supporting TDM-guided beta-lactam therapy is increasing, but the quality of evidence is still not convincing, and the findings of individual clinical studies are often contradictory^3,9^.

Five systematic reviews and meta-analyses of clinical research papers focusing on this topic have been published in the past years in an effort to synthesize and judge the available evidence, but all of these suffer from important limitations^10–14^. These include the consideration of only one subtype of these medications (penicillins^10^ or carbapenems^11^), the joint evaluation of therapies employing various antiinfectives^13,14^, assessment in heterogenous patient populations (critically as well as non-critically ill, adult as well as pediatric patients)^11–13^, and the inclusion of both non-peer-reviewed and peer-reviewed reports in the meta-analyses^10,12^. The translation of the outcomes to clinical practice is also impaired by the fact that none of the earlier meta-analyses have made any distinction among the various pharmacokinetic-pharmacodynamic (PK/PD) targets considered by the studies included, leaving clinicians without any specific guidance on which of these should be attained to achieve favorable progress.

Given the above limitations, a systematic review and meta-analysis with a narrow definition of the population, intervention, comparison, and outcomes (PICO) framework, as well as of the PK/PD target, can facilitate the clinical implementation of adjusted beta-lactam dosing protocols in adult critical care. The present work focuses strictly on the evaluation of peer-reviewed clinical investigations. In line with the recommendations of recent professional guidelines, the PK/PD target to attain is defined as the proportional period in which the concentration of the unbound fraction of the antiinfective substance exceeds the in vitro minimal inhibitory concentration (100%*f*T > MIC). Sub-group analyses are conducted whenever enough data is available to compare the results of RCTs to those obtained in observational studies. We have elaborated the hypothesis that this focused evaluation allows the inference of practical conclusions concerning intravenous beta-lactam therapy in critically ill adults. In accordance with our hypothesis, the population comprised critically ill adult patients receiving beta-lactam antibiotics (P). The intervention was a modification of the beta-lactam dosing regime based on TDM results (I), and the comparator was the application of standard care (C). The outcomes (O) are ranked based on the strength of their relationship with beta-lactam therapy.

## Results

### Characteristics of the studies included

**Figure 1:**
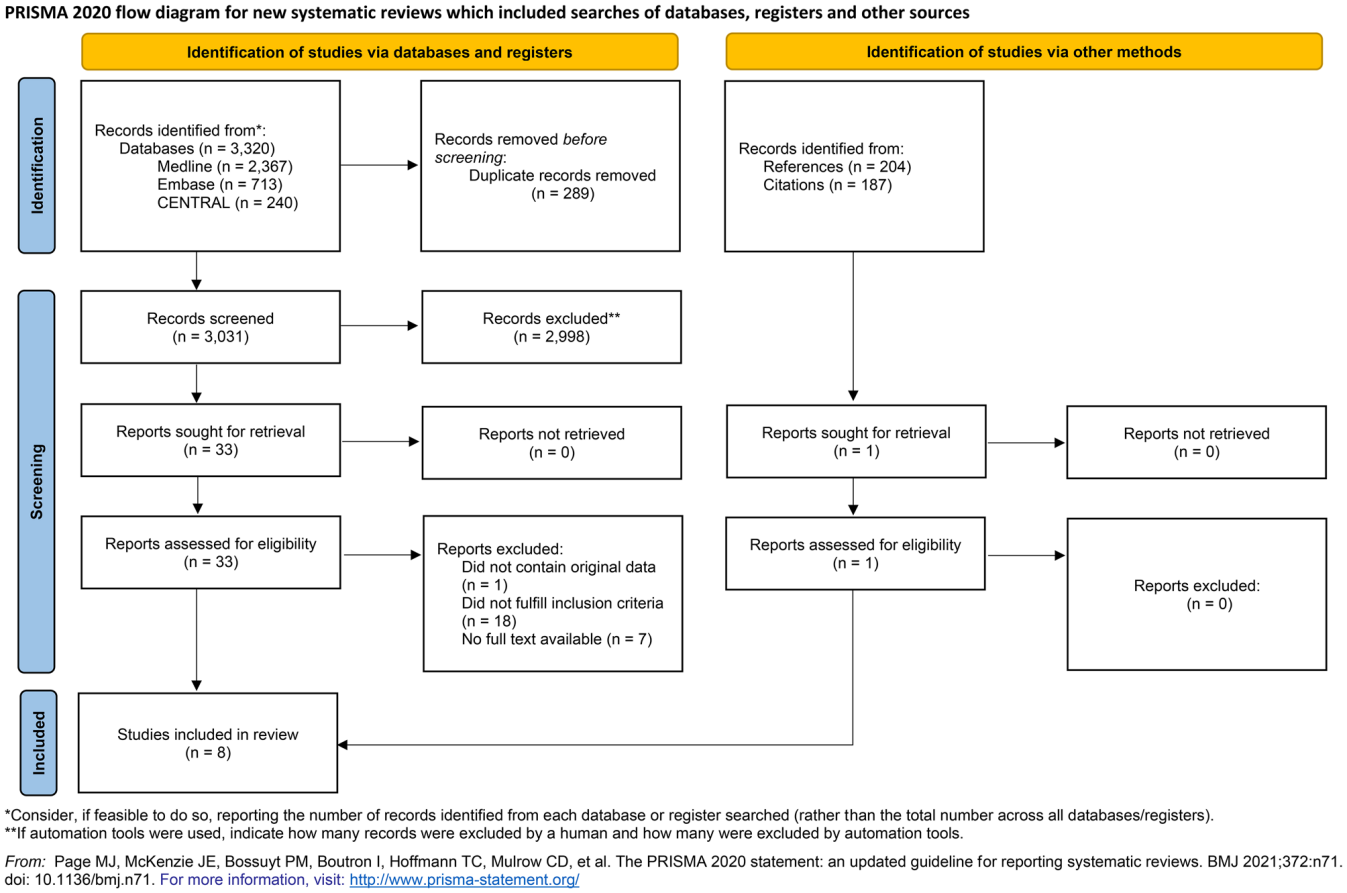
PRISMA flowchart showing the results of the literature database search.

The database searches yielded 3320 records, 33 of which were retrieved for full-text selection. The inter-rater agreement was excellent (Cohen's κ = 0.9). Seven articles (two randomized controlled trials^15,16^ and five retrospective observational studies^17–21^) were found eligible for evaluation (κ = 0.7). One prospective randomized, controlled trial was also found suitable based on the subsequent manual screening of the reference list^22^ (Fig. 1). The total number of patients was 1044. Six of the eligible works were single-center, and two were multi-center studies. We summarize the baseline characteristics of the articles included in Table 1. There was no overlap between any two populations in the meta-analysis.

**Table 1:** Characteristics of the included studies. *f*T > MIC and *f*T > 4xMIC, the proportion of the dosing interval in which the unbound concentrations of the antibiotic exceeded the minimal inhibitory concentration, or four times the minimal inhibitory concentration, respectively.

Author and year of publication	Type of study	Level of medical care	Population	Intervention	Control	Antibiotic type and MIC	Pharmacokinetic–Pharmacodynamic target	Extracted outcomes
De Waele et al. 2013^15^	RCT	Medical and surgical ICU of Ghent University Hospital, Belgium	Antibiotic treatment with PTZ and/or MEM with normal renal function	Daily TDM with dose adjustment as needed	Conventional treatment: daily TDM, but the physician was blinded to result	PTZ and MEM extended infusion	100% *f*T > MIC; 100% *f*T > 4 × MIC	Clinical cure microbiological eradication target attainment: 100% *f*T > MIC 100% *f*T > 4 × MIC treatment failure in-hospital mortality 28-day mortality ICU mortality
			n = 41	n = 21	n = 20	MIC: epidemiological cutoff value of wild-type *Pseudomonas* species		
Fournier et al. 2018^22^	RCT	Lausanne Burn Intensive Care, Switzerland	Burn trauma patients receiving intravenous antibiotics	Real-time TDM and online antibiotic adaptation	Dose adjustment upon clinician’s discretion	Intermittent bolus, various beta-lactams	Trough level > MIC, or above a predefined concentration	Clinical cure treatment failure ICU mortality ICU length of stay
			n = 38	n = 19	n = 19	MIC: actual MIC of isolated pathogen or EUCAST clinical breakpoint for *P* *aeruginosa* and Enterobacteriaceae		
Hagel et al. 2022^16^	RCT	13 ICU centers in Germany	Patients with severe sepsis or septic shock	Dosing of piperacillin/ tazobactam was guided by daily TDM	Piperacillin/ tazobactam was administered without use of TDM	Continuous infusion, piperacillin/ tazobactam	100% *f*T > 4 × MIC	Clinical cure microbiological eradication target attainment: 100% *f*T > 4 × MIC treatment failure 28-day mortality ICU length of stay hospital length of stay
			n = 249	n = 125	n = 124	MIC: actual MIC of isolated pathogen		
Fournier et al. 2015^20^	RCS	Burn ICU Vaud, Switzerland	Patients admitted to the burn ICU receiving carbapenems	Patients with real-time TDM and adjusted dosages	Patients without TDM	Intermittent bolus, meropenem and imipenem	Trough concentration > MIC, upper trough limit of 8 mg/L; 100% *f*T > MIC	ICU mortality ICU length of stay
			n = 109	n = 27	n = 82	MIC: of causative organism; if no organism isolated, MIC of 1 mg/L; later, this was changed to 2 mg/L (per EUCAST)		
McDonald et al. 2016^17^	RCS	Tertiary referral ICU, Brisbane, Australia	Patients with suspected or confirmed infection with either MEM or PTZ regardless of organ function	‘High-dose group’	‘Licensed-dose group’	Infusion: intermittent bolus, MEM or PTZ continuous or extended infusion in high-dose group to achieve target concentration	100% *f*T > MIC	Clinical cure microbiological eradication target attainment: 100% *f*T > MIC treatment failure in-hospital mortality ICU length of stay hospital length of stay
			n = 93	n = 25 MEM group n = 23 PTZ group	n = 22 MEM group n = 23 PTZ group	MIC: EUCAST clinical breakpoint		
Aldaz et al. 2021^18^	RCS	Clínica Universidad de Navarra, Pamplona, Spain	Critically ill patients receiving meropenem	Patients who received meropenem dose adjusted by TDM	Patients who received meropenem adjusted following standard recommendations	Extended infusion, meropenem	*f*T > 4 × MIC	Clinical cure microbiological eradication in-hospital mortality 14-days mortality ICU length of stay hospital length of stay
			n = 154	n = 77	n = 77	MIC was determined in each case when a pathogen could be identified. For empirical treat- ments and when the MIC was not available, 1 mg/L was used		
Nikolas et al. 2021^21^	RCS	University Hospital Wuerzburg, Germany	Critically ill patients receiving PTZ	With TDM	Without TDM	continuous infusion, piperacillin/ tazobactam	Target of total piperacillin concentration: 20 mg/L if MIC ≤ 4 mg/L; 40 mg/L if 4 mg/L < MIC ≤ 8 mg/L; 80 mg/L if 8 mg/L < MIC ≤ 16 mg/L, or pathogen is unknown	ICU length of stay
			n = 160	n = 114	n = 46	MIC: not reported		
Kunz Coyne et al. 2022^19^	RCS	UF Shands Gainesville and UF Health Jacksonville academic medical center, USA	Critically ill patients with *Pseudomonas* *aeruginosa* pneumonia and bloodstream infections receiving beta-lactams*	Routine beta-lactam TDM	Nonroutine beta-lactam TDM	Extended or continuous infusion of beta-lactams	100% *f*T > MIC; 100% *f*T > 4 × MIC	Clinical cure treatment failure in-hospital mortality ICU length of stay hospital length of stay
			n = 200	n = 95	n = 105	MIC: Clinical and Laboratory Standards Institute (CLSI) breakpoint		

*EUCAST* European Committee on Antimicrobial Susceptibility Testing, *ICU* intensive care unit, *MEM* meropenem, *MIC* minimal inhibitory concentration, *PTZ* piperacillin/tazobactam, *RCS* retrospective cohort study, *RCT* randomized clinical trial.

*cefepime, ceftazidime, ceftazidime/avibactam, aztreonam, meropenem and piperacillin/tazobactam.

### Primary outcomes

#### PK/PD target attainment

**Figure 2:**
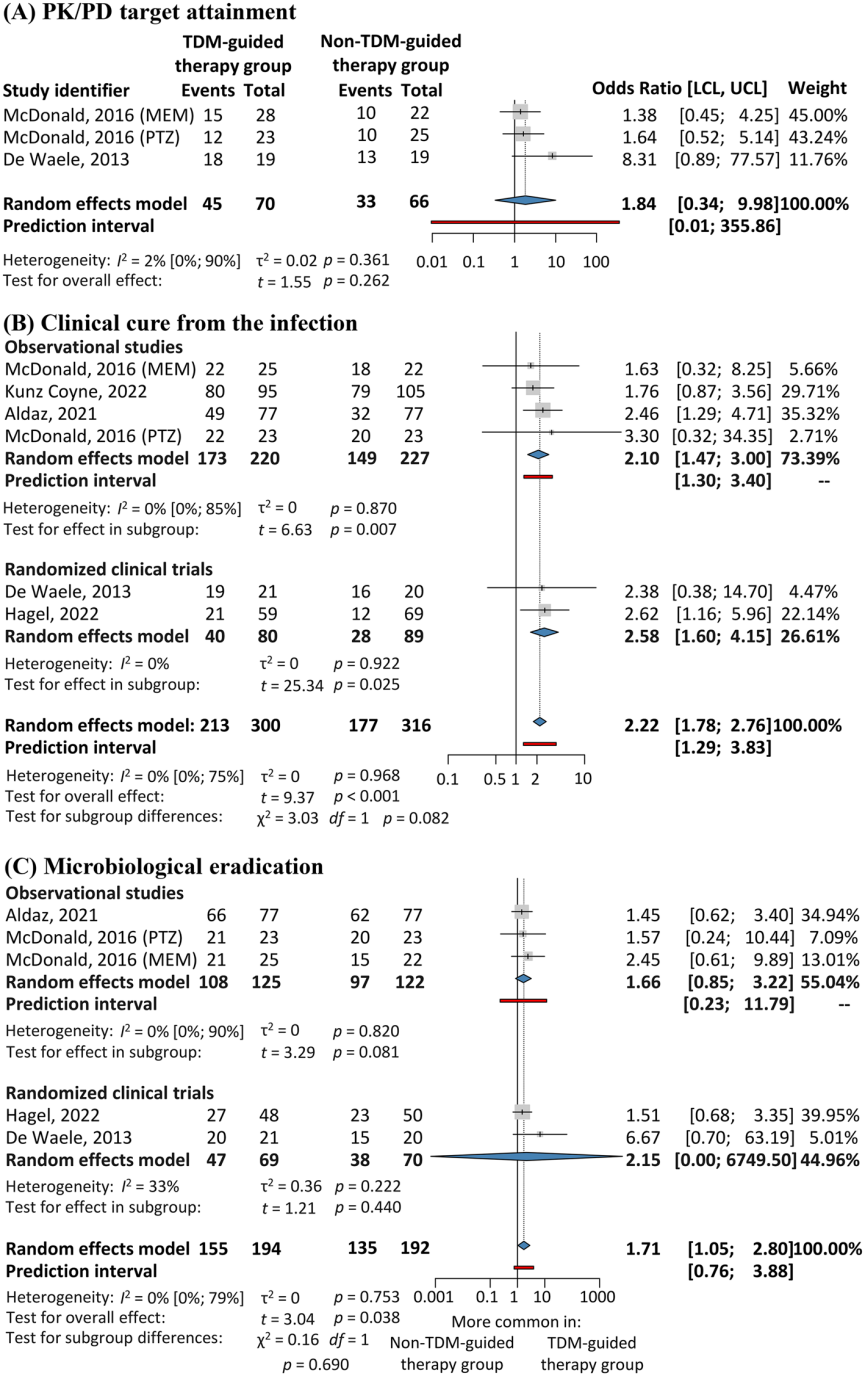
Evaluation of primary outcomes. (**A**) PK/PD target attainment, (**B**) clinical cure from the infection, and (**C**) microbiological eradication. LCL and UCL, lower and upper limits of the confidence interval. TDM, therapeutic drug monitoring.

The PK/PD target employed in the studies evaluated was the proportion of the dosing interval (fT) in which the unbound concentration of the antibiotic exceeded the minimal inhibitory concentration (MIC). The target considered was 100% *f*T > MIC, based on recommendation #26 of the Surviving Sepsis Campaign guideline^2^. The authors could extract data from two publications, comprising 136 patients^15,17^. We found no significant difference between the intervention and the comparison groups regarding the attainment of the PK/PD indices [OR 1.84; 95% CI 0.34–9.98; *I*^2^ = 2%, Fig. 2 (A)]. In two studies, the PK/PD target was 100% *f*T > 4xMIC^16,18^. In another publication, both 100% *f*T > MIC and 100% *f*T > 4 × MIC were considered as targets, and no sufficient data were provided for the comparison group that could have allowed their extraction^19^. In three publications, multiple PK/PD targets were employed^20–22^ (Table 1).

#### Clinical cure from the infection

Data on clinical cure from the infection were extracted from five studies including 616 patients^15–19^. Significantly higher odds of clinical cure were identified in the intervention group in the analysis of the pooled results [OR 2.22; 95% CI 1.78–2.76; *I*^2^ = 0%], as well as in the subgroup analysis of the observational studies [OR 2.10; 95% CI 1.47–3.00; *I*^2^ = 0%] and of the randomized controlled trials [OR 2.58; 95% CI 1.60–4.15; *I*^2^ = 0%, Fig. 2 (B)]. The definitions of clinical cure from the infection, in addition to those of other outcomes, were different among the evaluated studies (Table 2).

**Table 2:** Definitions employed in the publications included for evaluating clinical cure, microbiological eradication, treatment failure and/or improvement in clinical status.

Author and year of publication	Definitions of outcomes
De Waele et al. 2013^15^	Clinical resolution: disappearance of all signs and symptoms associated with infection Improvement: marked or moderate reduction in severity and/or number of signs and symptoms of infection Failure: insufficient lessening of signs and symptoms of infection to qualify as improvement, including death Microbiological eradication: response to therapy was also evaluated by bacterial persistence on day 7
Fournier et al. 2018^22^	Clinical cure: ND Improvement: ND Failure: ND Microbiological eradication: ND
Hagel et al. 2022^16^	Resolution: disappearance of signs and symptoms and no addition of antimicrobial therapy and no requirement for additional antibiotic treatment (except as part of de-escalation strategy) for the disease to be examined AND no initiation of antibiotic treatment for the disease to be investigated within 48 h after completion of the study drug Improvement: marked to moderate improvement in signs and symptoms and no addition of antimicrobial therapy and no initiation of antimicrobial therapy in 48 h after cessation of antibiotics Failure: signs and symptoms of infection persist or increase in comparison to baseline, or additional antibiotic treatment becomes necessary for the disease to be investigated Microbiological eradication: documented: elimination of the putative pathogen from repeated cultures of the site of infection presumed: disappearance of acute signs and symptoms related to the infection and no culture results available
Fournier et al. 2015^20^	ND
McDonald et al. 2016^17^	Resolution: antibiotic cessation due to microbiological control or de-escalation to narrower spectrum antibiotic triggered by clinical improvement and new microbiology data Treatment failure: escalation of antibiotic therapy with additional agents
Aldaz et al. 2021^18^	Clinical remission: absence of all signs and symptoms suggestive of infection including the normalization of temperature, C-reactive protein (CRP) and procalcitonin (PCT) levels in the absence of known sepsis markers. Normal CRP and PCT levels were defined ≤ 0.5 mg/L and ≤ 0.5 ng/mL, respectively Microbiological remission: cultures with no bacterial growth
Nikolas et al. 2021^21^	ND
Kunz Coyne et al. 2022^19^	Clinical cure: absence of all-cause in-hospital mortality, escalation and/or addition of antimicrobial therapy for *Pseudomonas aeruginosa* infection after 48 h of treatment due to worsening clinical status or transfer to a higher level of care Microbial eradication: eradication of *P. aeruginosa* from the index positive culture source up to hospital discharge when confirmed by ≥ 1 repeat culture. In cases where there were no repeated cultures and the patient had infection resolution, microbial eradication was assumed

Other outcomes are clarified in Table 1. ND, not defined in the publication specified.

#### Microbiological eradication

Data on microbiological eradication due to beta-lactam treatment could be extracted from four studies involving 386 patients^15–18^. The microbiological eradication rate was significantly higher in the intervention group in the pooled analysis [OR 1.71; 95% CI 1.05–2.80; *I*^2^ = 0%, Fig. 2C], but not in the sub-group analysis of observational studies or randomized controlled trials [OR 1.66; 95% CI 0.85–3.22; *I*^2^ = 0%, and OR 2.15; 95% CI 0.00–6749.50; *I*^2^ = 0%, respectively]. Various definitions were used in the publications evaluated (Table 2).

### Secondary outcomes

#### Treatment failure

**Figure 3:**
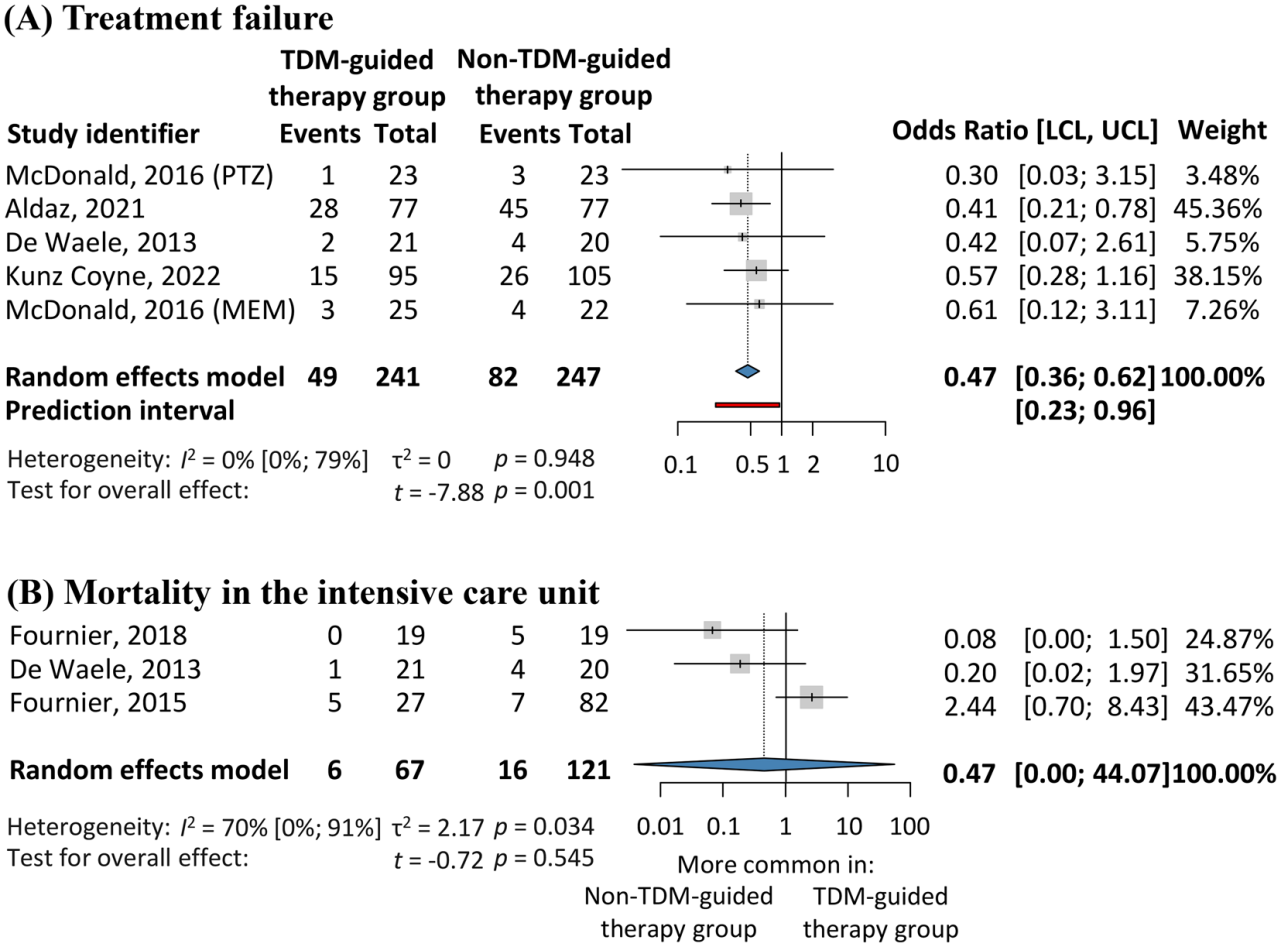
Secondary outcomes of the evaluation. (**A**) treatment failure, (**B**) mortality in the intensive care unit. LCL and UCL, lower and upper limits of the confidence interval. TDM, therapeutic drug monitoring.

A pooled analysis of 488 patients revealed significantly higher odds of treatment failure in the comparison group than in the intervention group [OR 0.47; 95% CI 0.36–0.62; *I*^2^ = 0%, Fig. 3A]^15,17–19^. Relevant data could not be extracted from the work by Hagel et al. who defined the lack of clinical cure as either improvement or treatment failure^16^.

#### Intensive care unit (ICU) mortality

Data on ICU mortality were extracted from three publications, including 188 patients. The pooled results revealed no significant difference between the interventionand comparison groups [OR 0.47; 95% CI 0.00–44.07; *I*^2^ = 70%, Fig. 3 (B)]^15,20,22^.

### Tertiary outcomes

#### In-hospital, 14-day and 28-day mortality

Data on in-hospital mortality were extracted from four articles including 488 patients^15,17–19^. We found a marginally non-significant difference between the two groups [OR 0.73; 95% CI 0.49–1.09; *I*^2^ = 79%]. Only one article (144 patients) reported on 14-day mortality. Two studies, including 290 patients, discussed 28-day mortality, with no significant difference between the intervention and comparison groups [OR 0.75; 95% CI 0.11–5.01; *I*^2^ = 79%]^15,16^.

#### ICU length of stay (LOS) and hospital LOS

All of the five observational studies [MD 6.66; 95% CI − 2.57 to 15.89; I^2^ = 82%]^17–21^ and two of the three RCTs [MD − 2.25; 95% CI − 12.67 to 8.18; I^2^ = 0%]^16,22^ reported on ICU LOS. The pooled analysis indicated no significant difference between the intervention and the comparison groups [MD 4.90; 95% CI − 1.96 to 11.75; *I*^2^ = 89%]. Four articles reported on hospital LOS, including 696 patients^16–19^. We found no significant difference between the two groups [MD 0.61; 95% CI − 3.65 to 4.87; *I*^2^ = 45%].

The results of the analysis of tertiary outcomes can be found in the supplementary information S4.

### Adverse events

Data could be extracted for comparing the occurrence of hematological and neurotoxic symptoms in the intervention and comparator populations. While there was no difference concerning the onset of neurological adverse effects [OR: 0.86; 95% CI 0.08–9.65; *I*^2^ = 38%], TDM-guided therapy was associated with a higher probability of developing hematological symptoms [OR: 1.55; 95% CI 0.93–2.58; *I*^2^ = 0%]. No sub-group analysis could be performed in this respect as all studies providing relevant data were observational (Supplementary information S5).

### Risk of bias assessment and quality of evidence

Overall, all results described in the RCTs were associated with a moderate risk of bias. The judgment was ‘some concerns’ mainly due to an inaccurate randomization process, and deviation from the intended interventions. The indicators ‘missing outcome data’, ‘measurement of the outcome’ and ‘selection of the reported result’ showed ‘low risk’ in all studies. The ROBINS-I tool yielded an overall ‘moderate risk of bias’ for observational studies, mainly due to the imprecise ‘classification of interventions’, ‘selection of the reported results’, and ‘selection of participants’. The ‘measurement of outcome’ conveyed a serious risk of bias regarding clinical cure from the infection in two studies^11,12^. ‘Confounding’, ‘deviation from intended intervention’ and ‘missing data’ showed low risk in the included works. The detailed results of the risk of bias assessment and the GRADE table are presented as Supplementary information S2, S3, S5.

## Discussion

### Principal findings

Five systematic reviews, four including a meta-analysis, have been published earlier to synthesize knowledge in this field. Nevertheless, none of them have focused exclusively on studies discussing the administration of intravenous beta-lactams, except for those focusing on a single subtype of beta-lactams, to critically ill adult patients, with the evaluation restricted to high-quality clinical research papers. Chronologically, Lechtig-Wassermann et al. were the first to synthesize knowledge on the impact of TDM-guided carbapenem therapy based on a literature search performed in December 2020. The outcomes of TDM-guided administration were compared to those observed when standard care was provided. Treatment with penicillins and cephalosporins was not considered. Mortality, the primary outcome, was lower in the intervention group [odds ratio: 0.75 (95% CI 0.49–1.13)]. The evaluation of secondary outcomes (morbidity, clinical cure, microbiological eradication, antimicrobial resistance, drug-related side effects, and the achievement of target concentrations) was based on two studies each. The quality of evidence was limited by the fact that one publication evaluated was a non-peer-reviewed conference abstract^10^.

In the subsequent related work by Luxton et al. RCTs, non-randomized cohort studies, and case studies discussing the TDM-guided administration of penicillins were reviewed by including investigations conducted with the participation of adult and pediatric critically ill patients (both with retained kidney function and with renal failure), non-critically ill patients treated with infections, as well as burn patients. In most publications included, the co-administration of various antibiotics occurred, therefore the relationship between a specific beta-lactam regime and the clinical outcomes was ambiguous. As many as sixteen PK/PD targets of one of three types (*f*T > MIC, *f*c_ss_ > MIC, or a specific target concentration) underwent a combined evaluation. No meta-analysis was performed due to the extreme heterogeneity and the overwhelmingly critical bias associated with the studies^11^.

A single systematic review and meta-analysis has focused on beta-lactam therapy, i.e. not a subtype of this group of substances, of the critically ill. The population comprised mainly adults and, in part, also pediatric patients. One study involved patients without evidence that all had received intensive care. Of the eleven publications found eligible, two were non-peer-reviewed conference abstracts. The updated results presented in one of these abstracts appeared in a subsequent peer-reviewed publication and is included in our analysis. The attainment of 50% *f*T > MIC and of 100%*f*T > MIC was assessed^12^.

In the meta-analysis of randomized clinical trials (RCT) by Sanz-Codina et al. the impact of TDM-guided treatment conducted with beta-lactams, ciprofloxacin, or vancomycin underwent combined evaluation^13^. Five studies we found eligible for our evaluation were not included^17–21^. A study we excluded due to the concatenation of data on therapies with beta-lactams, ciprofloxacin, and vancomycin, as well as an RCT based on the administration of piperacillin to non-critically ill febrile neutropenia patients were assessed^23,24^. PK/PD target attainment in the intervention and control groups was compared by considering all antibiotics and all targets. In a similar work, five RCTs focusing on the outcomes of treatment of critically ill adults with aminoglycosides, beta-lactams, ciprofloxacin, and vancomycin were evaluated^14^. The conclusions that TDM‑guided regimens were not beneficial in terms of clinical or pharmacological outcomes displayed a sharp contrast with those of Sanz-Codina et al. who found that PK/PD target attainment, treatment failure, as well as the risk of developing nephrotoxicity improved in patients subject to dose optimization. The joint evaluation of the attainment of three different PK/PD targets (100%*f*T > MIC, 100%*f*T > 4xMIC and AUC/MIC) was performed. Our evaluation of randomized and non-randomized clinical studies provides the most substantial evidence to date that defining and adjusting beta-lactam dosing regimens at adult intensive care units by considering systemic drug levels can be judged beneficial. Most importantly, clinical cure and microbiological eradication rates were significantly higher in the intervention groups. Hypothesis tests revealed no statistically significant difference in the attainment of PK/PD targets in the intervention and comparison groups; nevertheless, the proportions of patients who had attained the target were consistently higher in the intervention groups in the studies in which this outcome was evaluated. The odds ratio yielded by the random-effects model also supported this conclusion. TDM guidance has no impact on ICU mortality, but it does have a favorable impact on treatment failure. These results provide further evidence that, although TDM-guided beta-lactam antibiotic therapy has no impact on patient survival, it influences the success rate of overcoming an infection.

It is important to note that negative consequences, such as an increase in mortality or the development of serious adverse events (neurotoxic symptoms or renal failure), have never been associated with TDM-based therapies. Nevertheless, recent trials explicitly conducted to assess the clinical impact of TDM-guided treatments have yielded somewhat discouraging outcomes. The Right Dose, Right Now study, an investigator-initiated, two-center, randomized controlled, two-arm, paralleled, non-blinded superiority trial, compared the clinical status of 132 critically ill adult patients receiving antibiotics based on bedside, real-time, data-driven dosing calculations to that of 120 patients receiving standard care. The antibiotics monitored included ceftriaxone and meropenem. No significant difference was observed in achieving primary or secondary outcomes^23^. DOLPHIN, a multi-center, open-label, randomized trial involving eight academic and teaching hospitals in the Netherlands and 388 critically ill adult patients, of whom 189 received beta-lactam or ciprofloxacin doses based on model-informed precision dosing, and 199 received standard care, showed no reduction in the ICU length of stay, a crucial source of the financial burden of care^25^.

These studies also concluded that real-time monitoring and feedback may be of fundamental importance for the timely modification of dosing regimens. Indeed, the devil seems to lie in the details of conducting the study and the monitoring, clinical evaluation, and therapeutic implementation of TDM results. Lack of availability of real-time TDM results led to failure to achieve PK/PD targets even in a significant proportion of critically ill patients receiving the antibiotic by continuous intravenous infusion^26^. On the other hand, in a multicenter, prospective, observational cohort study involving intensive care units in three tertiary facilities (BLAST 1), the PK/PD target itself was directly associated with clinical outcomes. Failure to achieve 40–50% *f*T > MIC within 48 h in meropenem and piperacillin regimes was significantly associated with all-cause mortality while achieving 100% *f*T > MIC within 48 h was significantly associated with shorter hospital stay^27^.

We consider the separate evaluation of observational studies and RCTs to strengthen our analysis. Unfortunately, sub-group analysis could not be performed to compare the outcomes of extended/continuous infusions versus therapies conducted by administering intermittent bolus doses of beta-lactams due to the availability of a single paper in which bolus dosing was employed consistently^20^. In two works, prolonged infusions with durations of 3–4 h were administered^15,18^. Fournier et al. applied a 30-min infusion from October 2013 to July 2015, while from August 2015 to October 2016, the infusion duration was increased to 2 h, starting from the second dose, due to an update of local protocols^22^. The sets of patients and outcomes could not be resolved. Hagel et al. employed a continuous infusion protocol^16^. Kunz-Coyne et al. applied three dosing schemes (30-min intermittent, extended or continuous infusion). In the intervention group, 72% of subjects received short intermittent infusions; in the comparator group, 95% received extended or continuous infusions. The type of intervention in the remaining 28% and 5% of patients, respectively, was not revealed by the authors^19^. McDonald et al. started with intermittent bolus dosing in all cases. They switched to prolonged infusion regimens in cases when the concentration of the unbound beta-lactam was lower than the target level by not more than 20%. These cases were nevertheless not presented in detail^17^. Finally, Nikolas et al. applied continuous infusions for 8 h or 12 h, followed by a wash-out period of the same duration^21^.

Performing the sensitivity analysis of the PK/PD target attainment assessment would have been useful for the investigation of the impact of the differences in targets considered in various publications. Unfortunately, only three data sets from two articles could be included in the evaluation of differences in target attainment. In three studies, the target was 100% *f*T > MIC, while two more studies were performed with targets of 100% *f*T > 4 × MIC, a meta-analysis of which could not be conducted. We decided that concatenating the two targets would yield similarly questionable outcomes as in the meta-analyses performed by others. Therefore, we included only the studies with a 100% fT > MIC target. Unfortunately, the differences in defining the MICs were retained as a statistical noise in the analysis.

The close similarities between our analysis and the one performed by Pai Mangalore et al. warrant a closer look at the differences between the two works. First, in the paper by McDonald et al. the resolution of infection was defined as antibiotic cessation due to microbiological control or de-escalation to a narrower spectrum antibiotic. We adhered to this definition, while Pai Mangalore et al. only considered cases in which microbiological control was established, excluding cases with de-escalation. Concerning the work by Aldaz et al. we considered the restoration of procalcitonin concentrations to a value within the reference range. Pai Mangalore et al. used data associated with the reduction in procalcitonin levels by at least 80%. Our approach is more appropriate as such a reduction rate does not necessarily yield a test result in the reference range (0–0.05 ng/mL). Of note, Aldaz et al. measured procalcitonin concentrations of 0.24–28.3 ng/mL and 0.26–29.0 ng/mL in the two patient groups, which exceeded the upper limit of the reference range 4.8–580 times, with medians of 4.58 ng/mL and 4.70 ng/mL, 91.6 and 94.0 times the upper limit of the reference range, respectively. Despite the profound differences in approach, Pai Mangalore et al. also found that TDM-guided treatments were beneficial over standard care regarding PK/PD target attainment, clinical cure, and microbiological eradication^12,18^.

Although mortality was not significantly different between our intervention and comparison groups, the point estimate was considerably smaller (0.47) than the one calculated by Pai Mangalore et al. (0.85). The latter is noteworthy in view of the fact that Zeggil and Dalton later adjusted this odds ratio for mortality to 0.90 after including further data, and interpreted it as a value even closer to the null effect^28^.

TDM is not a specific intervention, and it must be emphasized that efficient clinical decision-making algorithms are indispensable for making rational interventions based on TDM results. None of the studies in our analysis employed model-informed precision dosing. Instead, decisions were based solely on the relation of trough concentrations measured or estimated to the PK/PD targets considered. De Waele et al. increased the frequency of dose administration when the concentration of the unbound fraction of the antibiotic was lower than 4xMIC. In the case of meropenem, a 50% dose increase followed when the target was still not attained. When the concentrations were higher than 10xMIC, the frequency of administration was decreased, but only in cases when the frequency of administration had been increased before. Otherwise, the dose was reduced^15^. Fournier et al. adjusted antibiotic dosages to meet the desired pharmacodynamic targets^20^. In their later work, these authors presented a chart-based algorithm containing dose changes as well as the number of doses given. Dose adjustment was feased based on the antibiotic concentrations measured, and on the actual dosing regime employed. Renal function was also evaluated before making a clinical decision^22^. Hagel et al. adjusted the drug dosages in response to the lack of target attainment, with their judgment also taking various clinical parameters into account^16^. Kunz-Coyne et al. described dose modifications and changing the infusion protocol as tools for attaining the PK/PD targets^19^. McDonald et al. modified the dosing frequency or the infusion protocol based on the concentration of the unbound antibiotic measured^17^. Finally, Nikolas et al. applied dose adjustment to attain the specific PK/PD targets^21^. In a study, the Sawchuk-Zaske method and, subsequently, nonparametric pharmacokinetic modeling were employed for constructing a population model of meropenem, which could be used for simulating the attainment of PK/PD targets in the patients involved retrospectively^18^.

There is a growing consensus that the antibiotic regimes of critically ill patients should be optimized and monitored by employing TDM. At the same time, well-established considerations have scarcely been raised against this approach. As the admission of patients to the ICU itself impairs the clinical prognosis, optimizating all therapeutic measures is crucial for improving the perspectives of patients and the cost-efficiency of care^29^. The authors of the Surviving Sepsis Campaign endorse treatment optimization based on pharmacokinetic-pharmacodynamic (PK/PD) indices with the involvement of a skilled clinical team, population-specific guiding documents, TDM, and, potentially, dosing software. For beta-lactams, the recommended PK/PD index is a trough concentration higher than the minimal inhibitory concentration (MIC) of the identified pathogen (c_min_ > MIC)^2^. A position paper published by five leading international societies on intensive care, antimicrobial therapy, and TDM emphasized that TDM should be the standard for treatment with antibiotics, including beta-lactams, at intensive care units^3^. Some argue that the quality of data supporting the utility of TDM in beta-lactam therapy needs to be improved to make straightforward recommendations^4^.

A detailed guideline has been set forth jointly by the French Society of Pharmacology and Therapeutics and the French Society of Anaesthesia and Intensive Care for optimizing beta-lactam regimes in the ICU. The PK/PD target proposed in this guideline is a trough concentration of the beta-lactam unbound to serum proteins 4–8 times the MIC. Performing the first beta-lactam TDM is recommended 24–48 h after the first dose, after any dose adjustment, in the event of a significant change in the condition of the patient, suspected pharmacokinetic variability or clinical signs of beta-lactam toxicity, or when initiating renal replacement therapy. The guideline recommends that the MIC should be determined by the microbiology laboratory. When the MIC of the isolated strain is unavailable, a critical epidemiological MIC covering all the MICs of wild-type strains is recommended. In Europe, this is an epidemiological cut-off proposed by the European Society of Clinical Microbiology and Infectious Diseases (EUCAST)^9^.

### Strengths and limitations

The main strength of this meta-analysis and systematic review is that the impact of TDM-guided beta-lactam treatments was assessed by including only carefully designed, peer-reviewed studies and focusing on the entire spectrum of beta-lactam medications administered in the ICU. A sub-group analysis, i.e., the separate evaluation of RCTs and observational studies, was also performed for the first time. Nevertheless, our evaluation has limitations. First, the number of studies included was low, which is remarkable given the everyday administration of beta-lactams in intensive care. The heterogeneity of the study design was also a significant limiting factor in terms of the credibility of statistical evaluation. Finally, data could not be extracted for evaluating other markers, e.g., the development of antimicrobial resistance.

The low number of clinical studies eligible for meta-analysis, the heterogeneity of the types of healthcare facilities involved, the differences among the patient populations involved, and the diversity of the dosage regimens applied warrant a critical interpretation of the results presented. The heterogeneity of the definitions of clinical cure in the included studies is of particular concern. Definitions such as disappearance of all signs and symptoms of the infection^15,16^, “resolution” of infection^17^, the combination of the absence of all-cause in-hospital mortality, escalation and addition of antimicrobial therapy after 48 h of treatment due to worsening clinical status or transfer to a higher level of care^19^, as well as the normalization of the body temperature in combination with C-reactive protein and procalcitonin concentrations lower than 0.5 mg/mL and 0.5 ng/mL, respectively^18^, have been employed by various researchers. As most authors collected different data types, comparing the outcomes has limited validity. Another source of evaluation bias is the weighting used in the statistical calculations by considering only the number of patients involved in each study without considering the content of the definition of clinical cure.

A significant limitation to performing the meta-analysis of the outcomes of PK/PD target attainment obtained in various studies is the lack of standardization regarding establishing unbound concentrations. Concerning the eight clinical research papers evaluated in previous meta-analyses and the present work in this respect, unbound drug concentrations were assayed directly in a single study^17^, with another one claiming, without any demonstration, that unbound levels had been measured^25^. In the rest of the investigations, total antibiotic concentrations were determined. Some authors considered a pre-defined percentage of the measured concentration to account for the unbound fraction^22,24,30^. In two studies, total concentrations were measured, yet unbound fractions were considered to evaluate target attainment without specifying the relationships between the two^15,16^. Finally, total concentrations were measured and included in calculating PK/PD indices in a single research^23^.

Finally, the selection of the random-effect modeling approach may warrant some explanation. Our analysis yielded no evidence that fixed-effect models could perform superior to random-effect models. By statistical theory, employing fixed-effect models could not be justified, as the homogeneity of the statistical population investigated, a requirement for the valid use of fixed-effect models, could not be assumed. Instead, we aimed to identify outcomes with significant differences between the intervention and the comparator groups comprising a heterogenous set of data, which could be conducted using random-effect models.

### Implications for research and clinical practice

Currently, the practice of TDM-guided beta-lactam therapies is not widespread. In fact, it is rarely available in ICU wards, even in countries with a high national income^31^. The profound significance of overcoming organizational and technical difficulties in successfully implementing TDM-guided treatments has recently been discussed by Ewoldt et al.^32^. The lack of formal agreements by management, and the absence of clear evidence of effectiveness and cost-effectiveness were identified as the key barriers. Moreover, several survey respondents reported that the information and resources required to apply TDM had been improperly supplied. It is reasonable to assume these factors have practical implications for clinical studies. In the future, standardization and registration of clinical study protocols would facilitate the comparison of outcomes.

Other factors, such as costs and human resources, also play an essential role in the quality of the clinical implementation of TDM-based therapies, requiring attention and the education of healthcare personnel in this respect. In addition, while all efforts aim to achieve an increased success rate of antibiotic treatments, this outcome may result in longer stays at the ICU and other healthcare facility units in a proportion of cases, increasing the organization’s financial and human resources burden. As a result, the complexity of TDM-guided antibiotic therapies stretches far beyond the difficulties associated with drug-level measurements and decision-making algorithms.

## Conclusions

This systematic review and meta-analysis has found that the TDM-guided administration of beta-lactam antibiotics benefits critically ill patients. TDM-guided beta-lactam treatment is associated with improved clinical cure from infection and microbiological eradication, and a lower probability of treatment failure. We found no significant association between TDM guidance and outcomes less directly associated with beta-lactam regimes, including ICU length of stay, ICU mortality, or hospital mortality. High-quality randomized controlled trials with larger sample sizes are needed to establish the TDM-guided dosing of beta-lactam antibiotics. At the same time, it is essential to remember that the emphasis should always be on the quantitative magnitude of the benefits of TDM-guided therapies to individual patients rather than on the outcomes of statistical tests.

## Methods

### Data sources and searches

This systematic review was performed according to the guidelines of the Cochrane Collaboration^33^. The results are reported based on the Preferred Reporting Items for Systematic Reviews and Meta-Analysis (PRISMA) statement^34^. The review protocol was registered in the International Prospective Register of Systematic Reviews (PROSPERO) database (registration number: CRD42021285188).

The systematic search was performed in the databases EMBASE, PubMed/MEDLINE and Cochrane Central Register of Controlled Trials (CENTRAL) initially on 4 November 2021 and updated on 15 October 2022. The search terms were related to beta-lactam antibiotics, critically ill patients, and TDM (full search keys are provided as Supplementary information S1). No restrictions or filtering options were applied. The reference lists of all included reports were screened for further eligible articles.

### Study selection

The EndNote X9 citation manager (Clarivate Analytics, Philadelphia, PA, United States) was employed to select the publications to be included. After the automatic and manual duplicate removal (E.G.), two investigators (I.L.H and E.G.) independently screened the retrieved records in two steps: first by title and abstract, and, subsequently, by full text. Cohen’s Kappa coefficient (κ) was calculated to evaluate the inter-rater agreement after each selection step. Disagreements were resolved by third party arbitration (G.B.K. and M.A.E.). Our inclusion criteria were based on the PRISMA statement and the guidelines of the Cochrane Collaboration^33,34^.

### Data collection

One investigator (G.E.) manually extracted the data from the eligible articles. These were further checked by a second investigator (G.B.K) to ensure proper data quality. The following data were extracted: study characteristics (first author, year of publication, country, and number of centers), population data (sample size, percentage of female participants, age, and diagnoses), beta-lactam dosages, route of administration, treatment duration, and outcomes. Microsoft Excel (Microsoft Corporation 2016, Redmond, Washington, USA) was used for data collection.

### PICO criteria

The population comprised critically ill patients aged 18 or over, receiving a single beta-lactam as in intravenous infusion. The intervention was the adjustment of the dosing regime to ensure attaining the PK/PD target of 100%*f*T > MIC. The comparison group received the beta-lactam without TDM, or with no adjustment based on TDM results. The primary outcomes (target attainment, clinical cure from infection and microbiological eradication) were endpoints which are all crucial as well as interdependent indicators of the success of antibiotic therapy. Treatment failure and ICU mortality were considered as secondary outcomes. In-hospital mortality, the ICU LOS and the hospital LOS were evaluated as tertiary outcomes as these indicators are related to the outcomes of antibiotic treatment ambiguously. Only randomized controlled trials, prospective cohort studies and comparative retrospective studies were eligible for evaluation. Cross-sectional studies, case series, case reports, and studies not published in full detail, or not undergoing peer-review, were excluded. Investigations including non-critically ill or pediatric patients or where the evaluation involved the co-administration of beta-lactams with antibiotics with other chemical structures were excluded.

### Statistical analysis

Data were synthesized using the recommendations of the Cochrane Collaboration. The meta-analysis was performed for each outcome reported in at least two articles. To evaluate the differences between the intervention and comparison groups, pooled odds ratios (ORs) and mean differences (MDs) were calculated for binary and continuous outcomes, respectively, along with the corresponding 95% confidence intervals (CI) using random-effects models. Statistical heterogeneity was assessed by the *I*^2^ statistics (< 30%—low, 30% to 60%—moderate, 50% to 90%—substantial, and 75% to 100%—considerable degree of heterogeneity). For *I*^2^ results, the threshold of statistical significance was *p* = 0.10, it was set to 0.05 for all other cases All analyses were carried out in R (version 4.1.3) [R Core Team, Vienna, Austria] using the *meta* and *dmetar* packages^35,36^.

### Risk of bias assessment

The risks of publication bias were assessed by two independent authors (E.G. and I.L.H.) using the Cochrane risk-of-bias tool (RoB2) and the Robins-I tool for RCTs and observational studies, respectively^37,38^. Disagreements were resolved by author consensus. The domains evaluated with RoB2 included the randomization process, deviations from the intended intervention, missing data, outcome measurement, and selection of the results reported. Each domain was evaluated by the investigators. The risk assessment was performed by the software tools employed. The overall risk of bias was characterized as (1) low, (2) of some concern, or (3) high.

### GRADE

The recommendations of the Grading of Recommendations, Assessment, Development and Evaluations (GRADE) were employed to assessthe level of evidence. Each outcome was rated for risk of bias, inconsistency, indirectness, and imprecision as (1) not serious, (2) serious or (3) very serious. Certainty scores were categorized as (1) very low, (2) low, (3) moderate or (4) high by the online GRADEpro tool^39,40^.

### Supplementary Information

Supplementary Information.

## Data Availability

Data are available in the supplementary material.
