# Supplementary material for: Assessment of the practical impact of adjusting beta-lactam dosages based on therapeutic drug monitoring in critically ill adult patients: a systematic review and meta-analysis of randomized clinical trials and observational studies
Source: Sci Rep. 2024 Apr 2;14:7793. doi: 10.1038/s41598-024-58200-w (PMC10987621; doi:10.1038/s41598-024-58200-w)
Supplement: Supplementary file 1 — Supplementary Information. [file 41598_2024_58200_MOESM1_ESM.docx]

**Supplementary information**

**S1. Search key:**

**Pubmed:**

(intensive care OR critically ill OR intensive care unit OR "icu" or "ICUs" or critical care OR sepsis OR septic
shock) AND (tdm OR drug monitoring OR pharmacokinetics OR pk/pd) AND (β-lactam OR beta-lactam OR
carbapenem OR cephalosporin OR monobactam OR penicillin OR benzylpenicillin OR dicloxacillin OR
flucloxacillin OR amoxicillin OR ampicillin OR piperacillin OR tazobactam OR cefazolin OR cefuroxime OR
ceftazidime OR ceftriaxone OR cefixime OR cefalotin OR cefotaxime OR cefepime OR ceftaroline OR
doripenem OR ertapenem OR meropenem OR imipenem OR cilastatin OR aztreonam)

**Central and Embase:**

("intensive care" OR "critically ill" OR "intensive care unit" OR "icu" or "ICUs" or "critical care" OR sepsis OR
septic shock) AND (tdm OR "drug monitoring" OR pharmacokinetics OR pk/pd) AND (β-lactam OR
beta-lactam OR carbapenem OR cephalosporin OR monobactam OR penicillin OR benzylpenicillin OR
dicloxacillin OR flucloxacillin OR amoxicillin OR ampicillin OR piperacillin OR tazobactam OR cefazolin OR
cefuroxime OR ceftazidime OR ceftriaxone OR cefixime OR cefalotin OR cefotaxime OR cefepime OR
ceftaroline OR doripenem OR ertapenem OR meropenem OR imipenem OR cilastatin OR aztreonam)

**S2. Risk of bias assessment - RoB2**

**
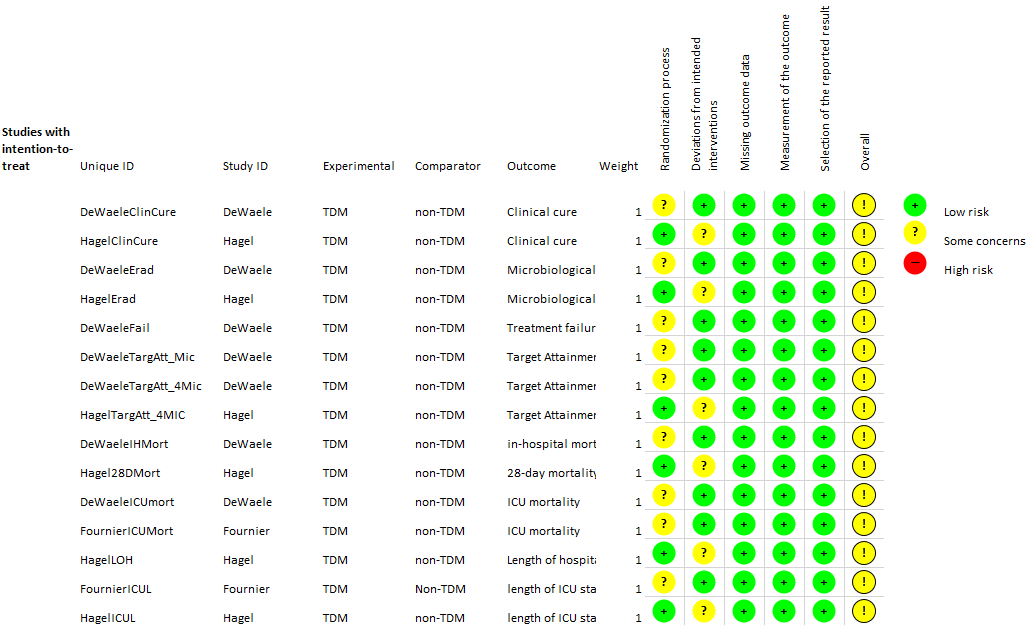
**

**S3. Risk of bias assessment- Robins-I**

**Clinical cure**


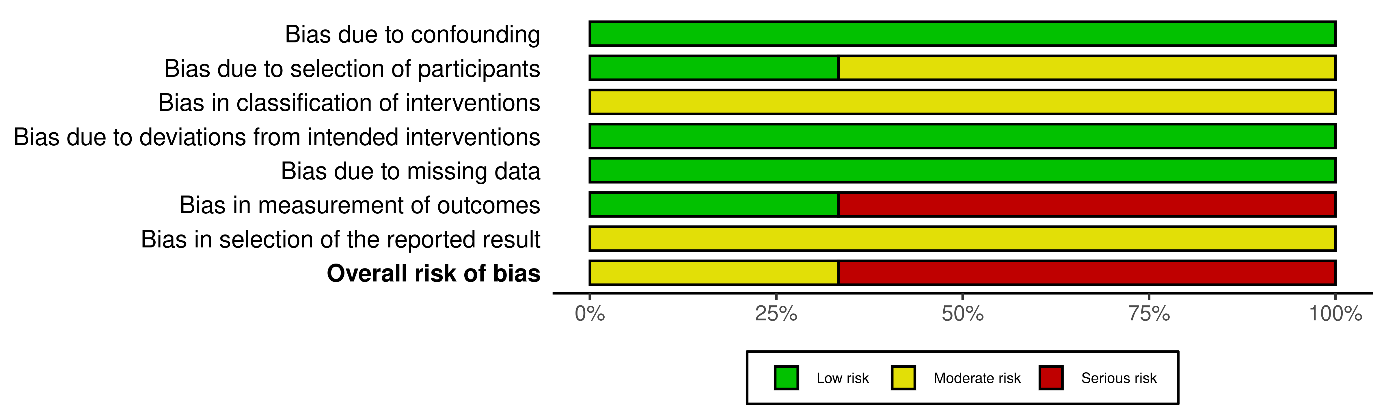

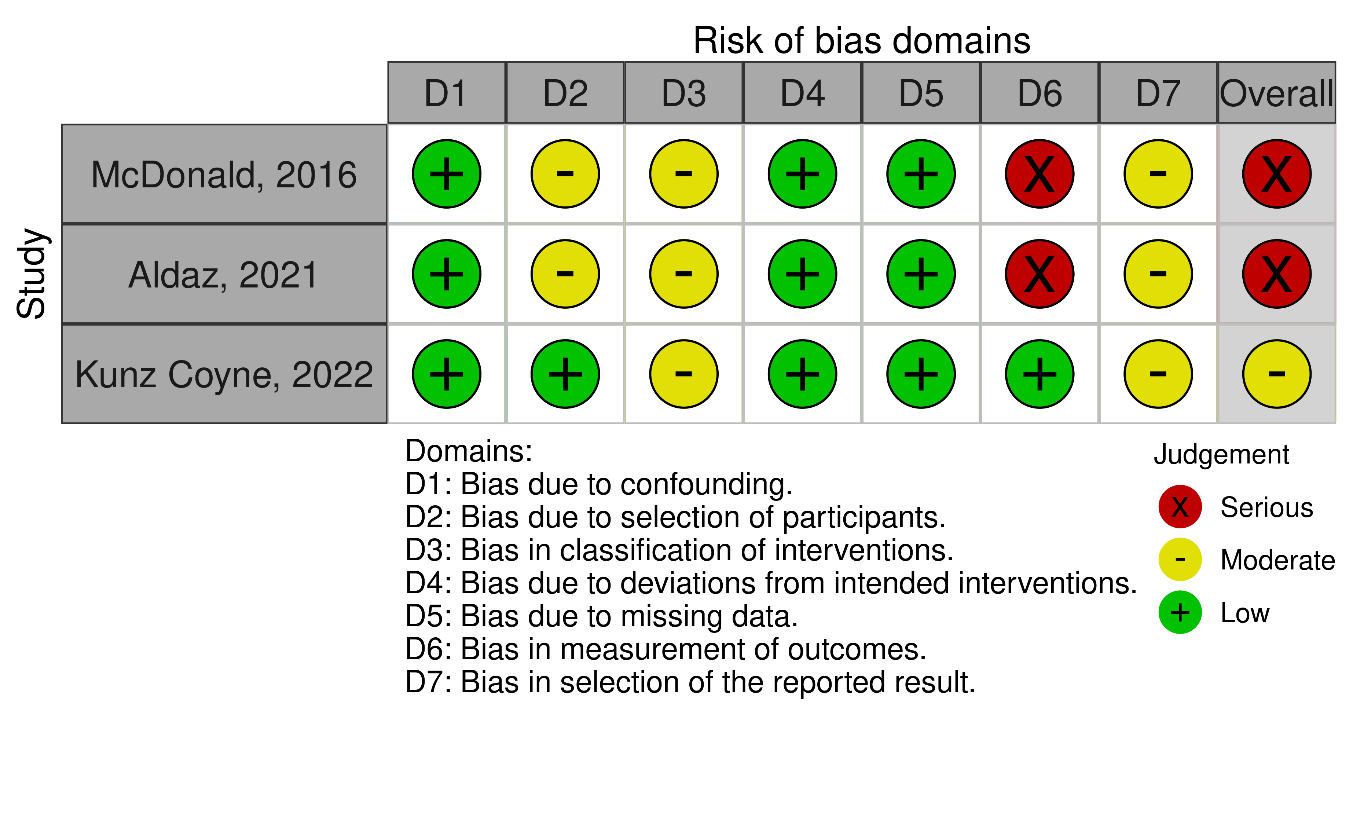


**Microbiological eradication**


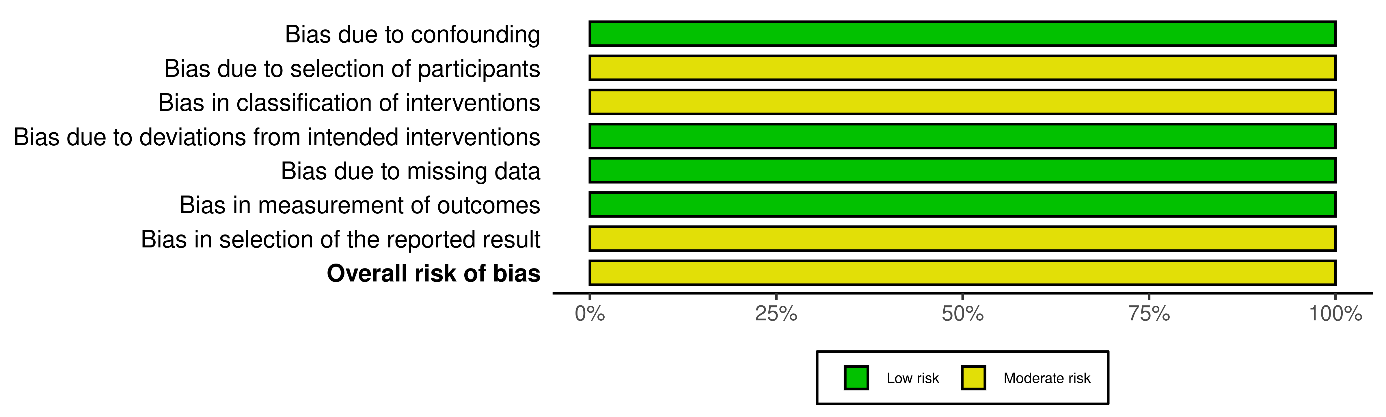

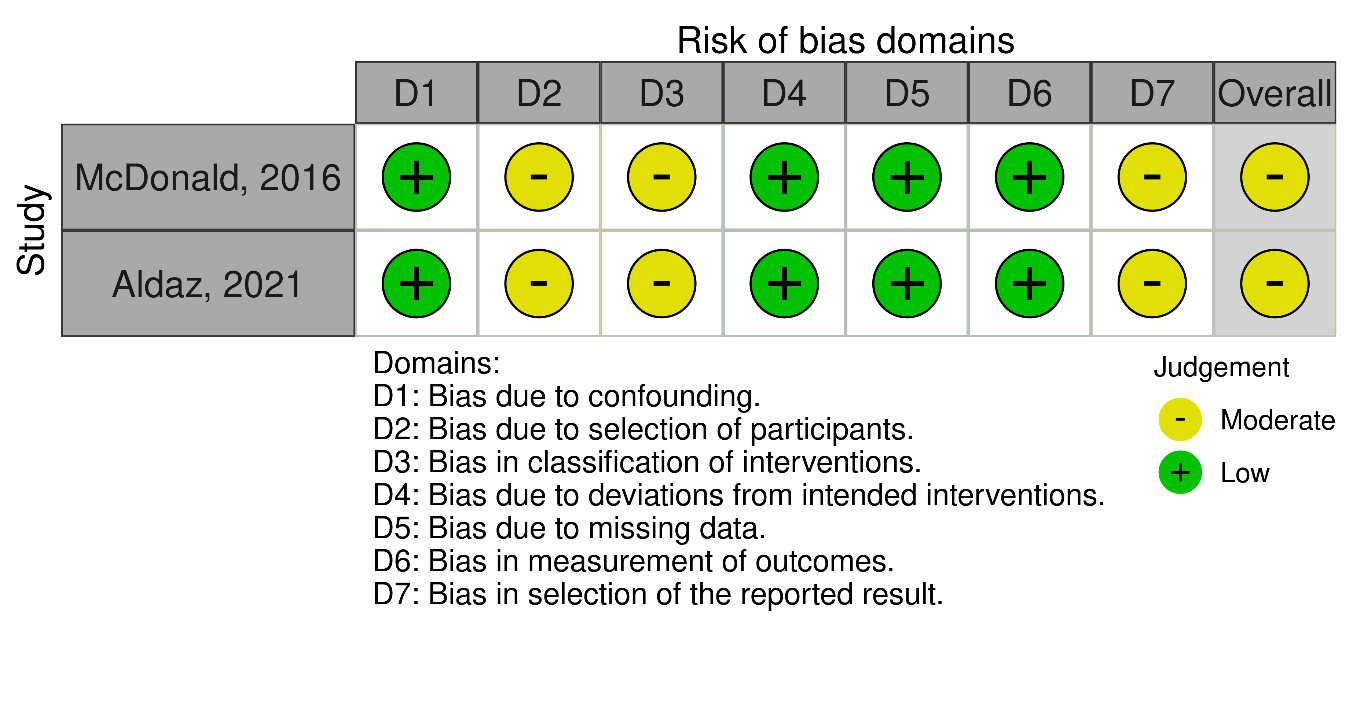


**Hospital mortality**

**
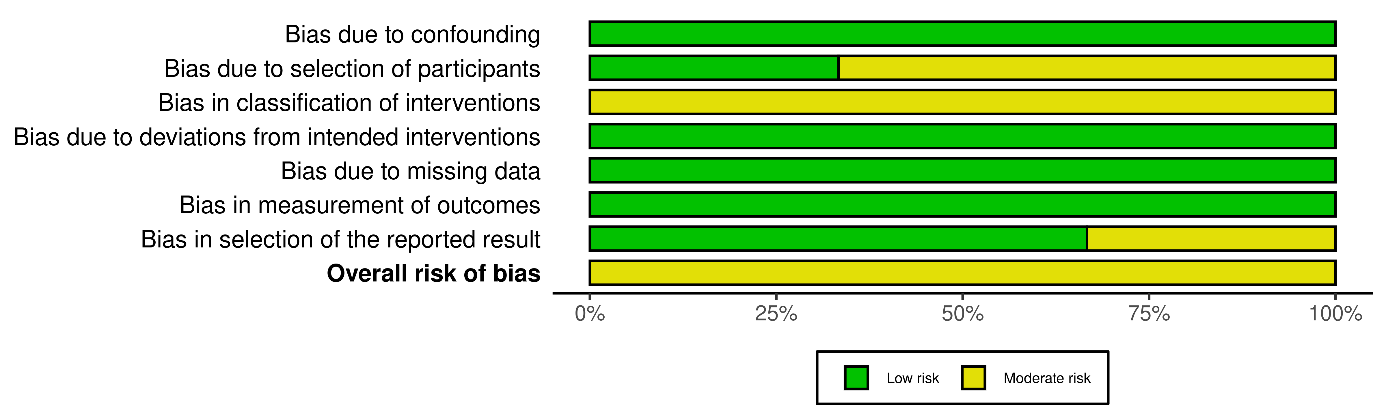

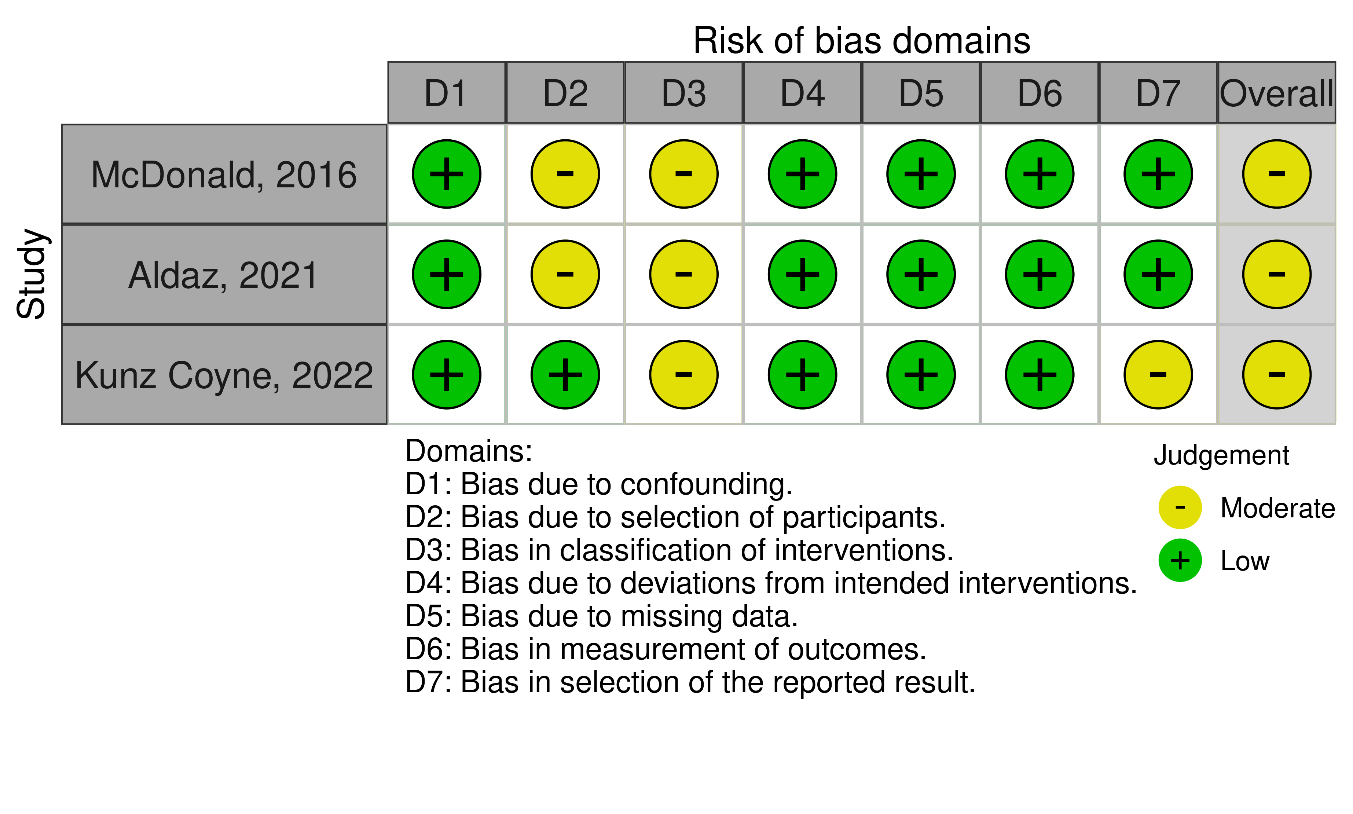
**

**Length of ICU stay**

**
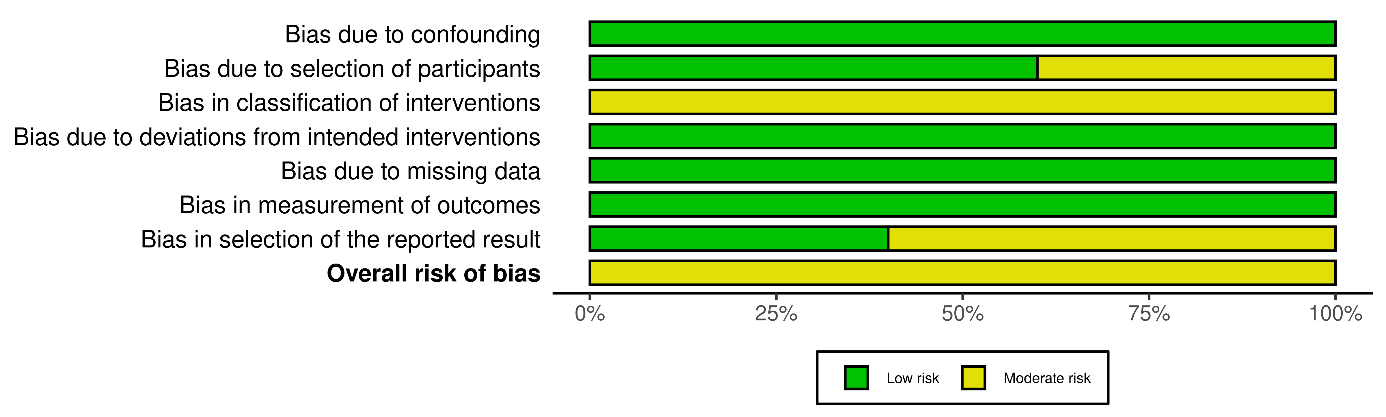

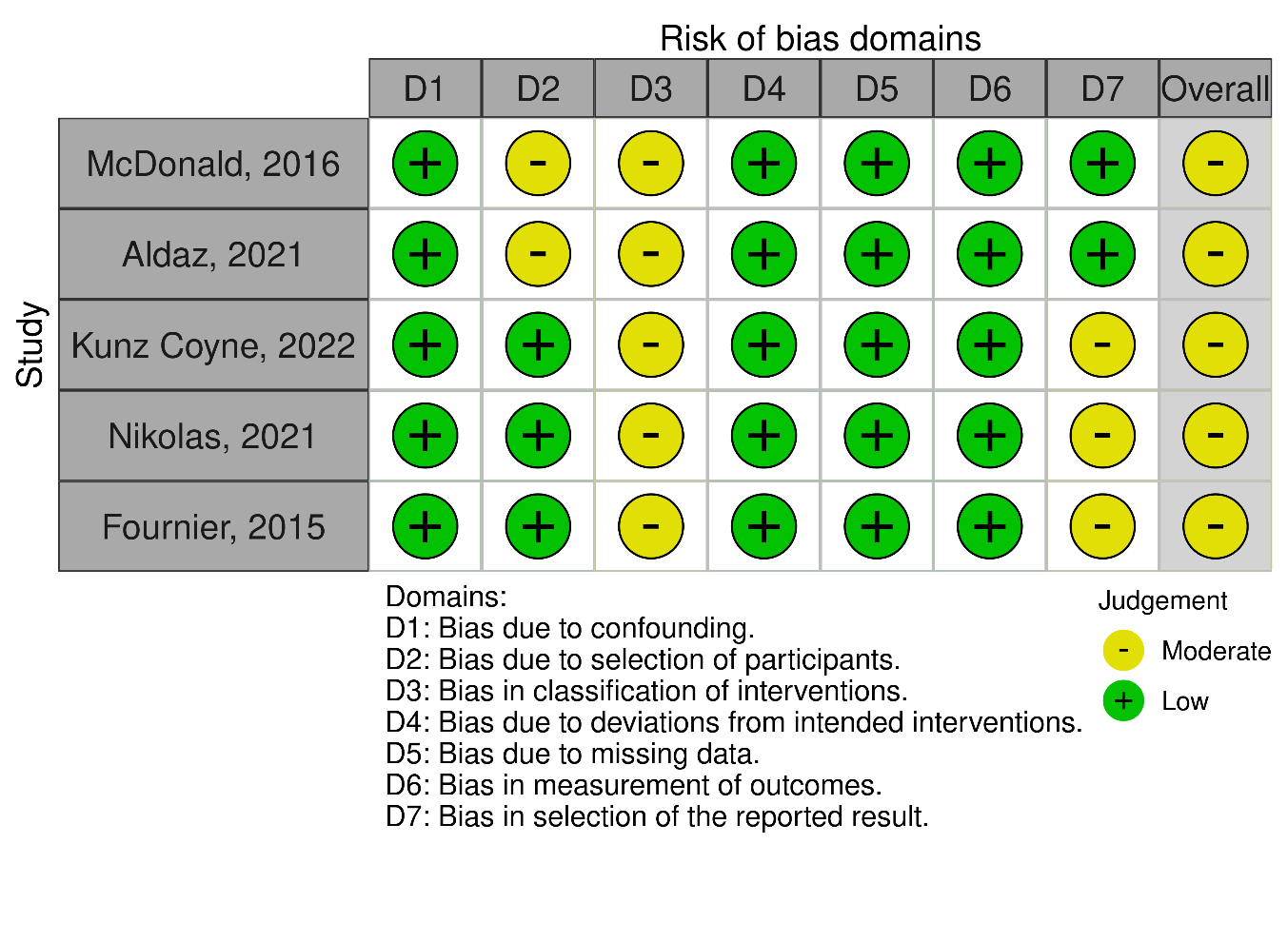
**

**Length of hospital stay**

**
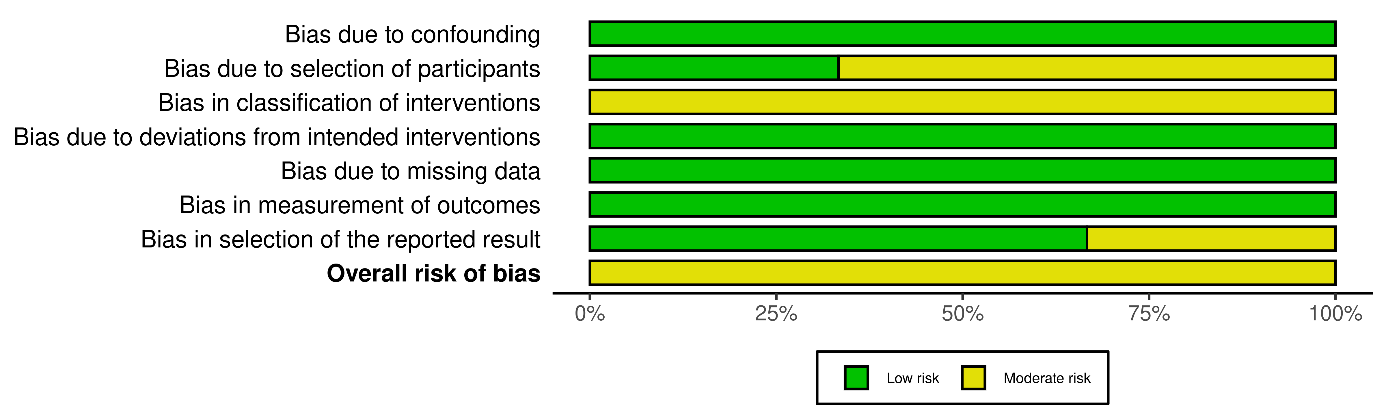

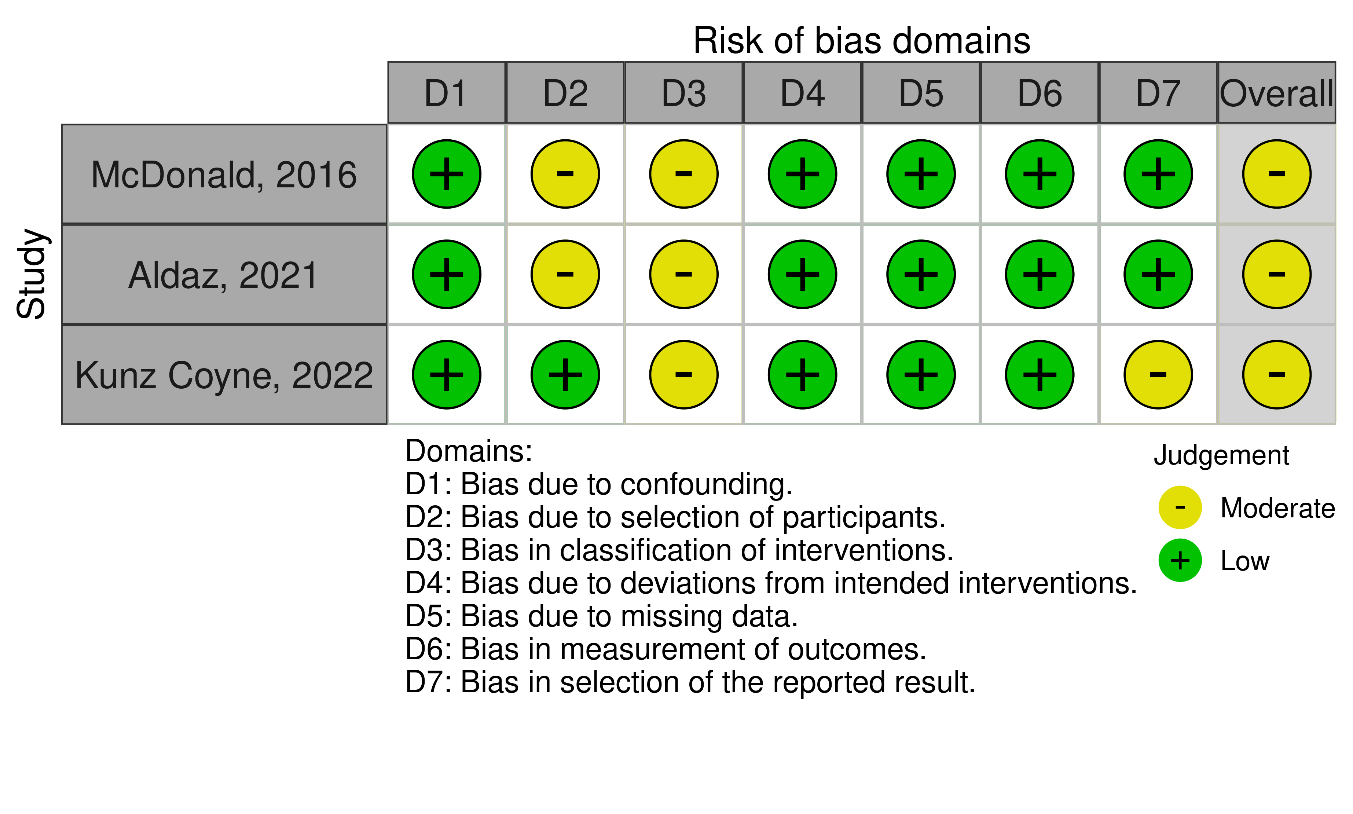
**

**Intensive care unit mortality**

**
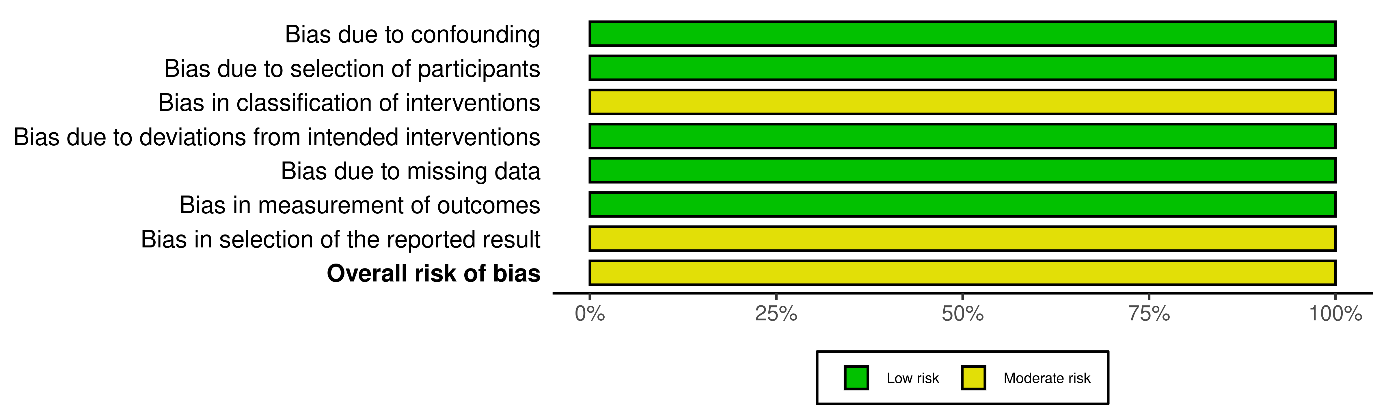

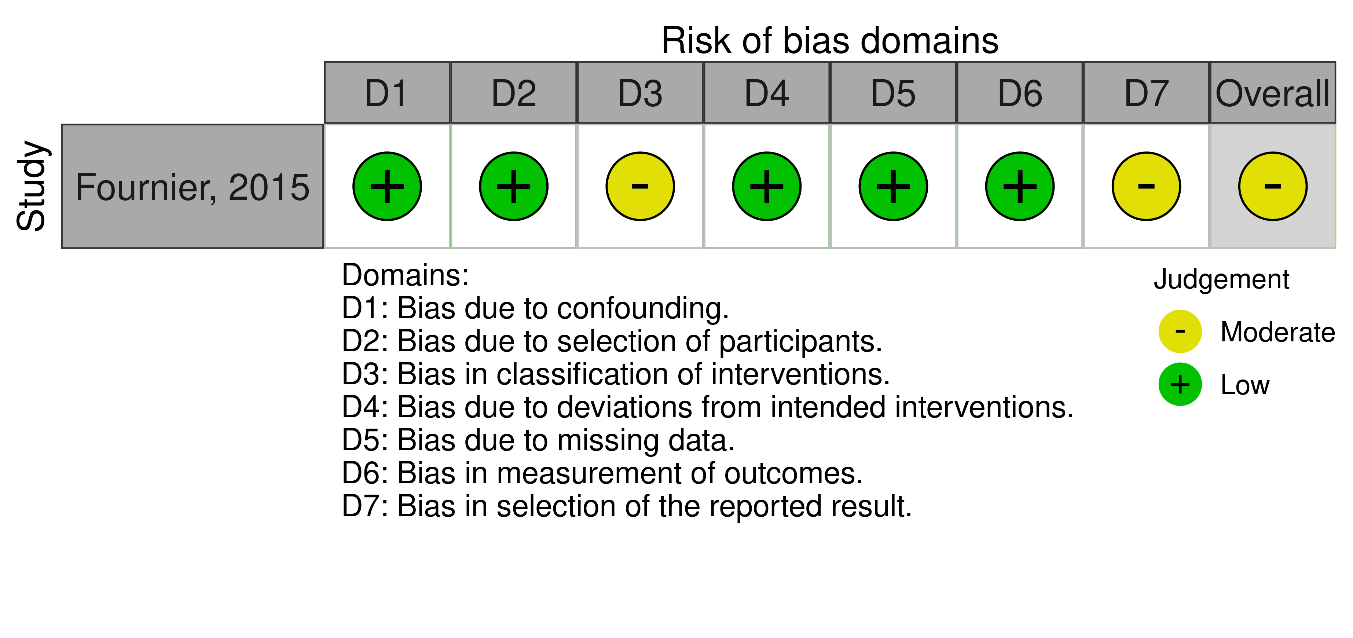
**

**S4. Tertiary outcomes**

**In-hospital mortality**

**
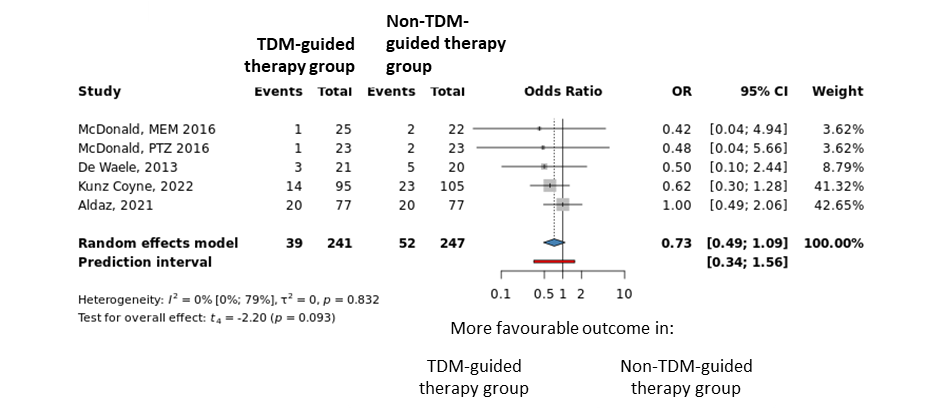
**

**28-day mortality**


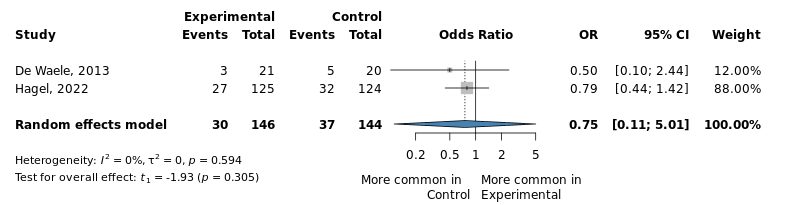

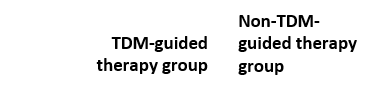

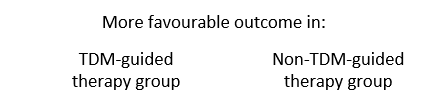


**Length of stay at the intensive care unit**


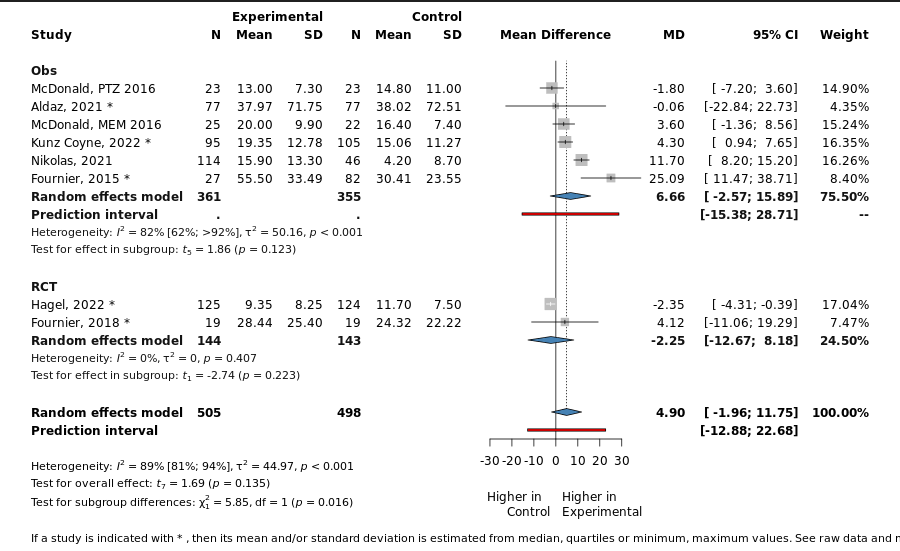

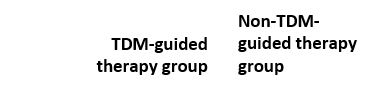

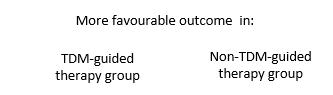


**Length of stay in the hospital**


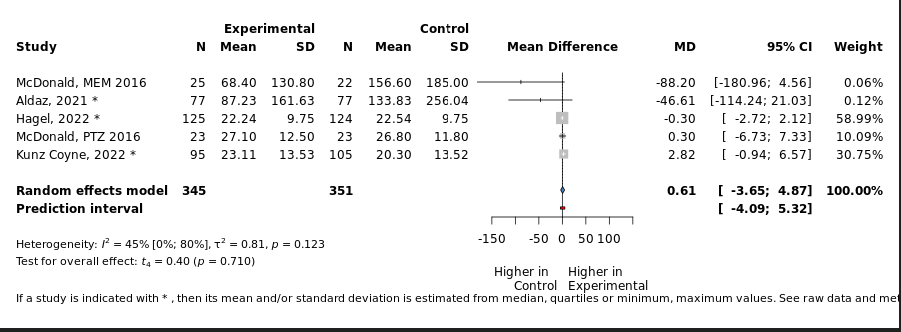

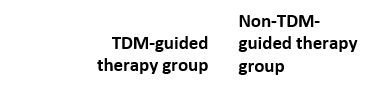

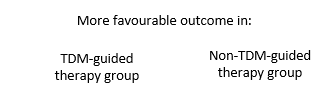


**S5. Adverse events**

**Development of haematological symptoms**

**
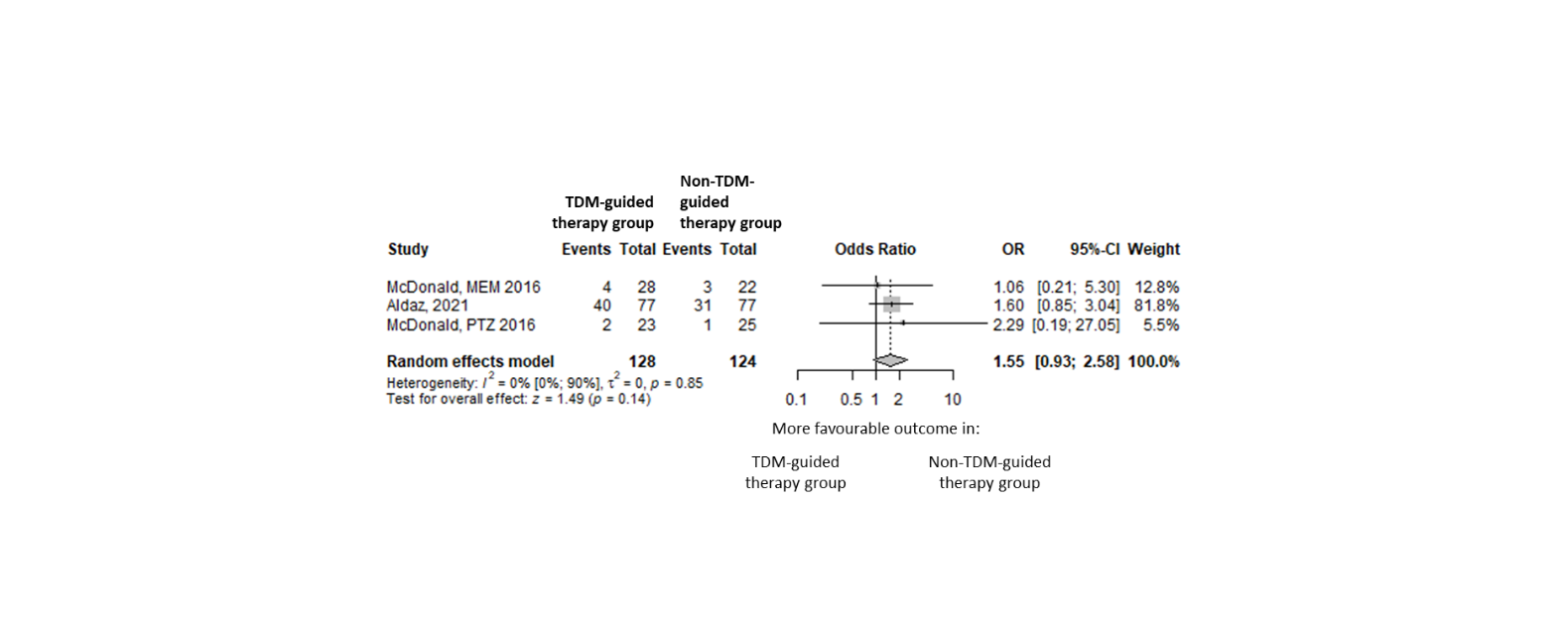
**

**Occurrence of neurotoxic symotoms**

**
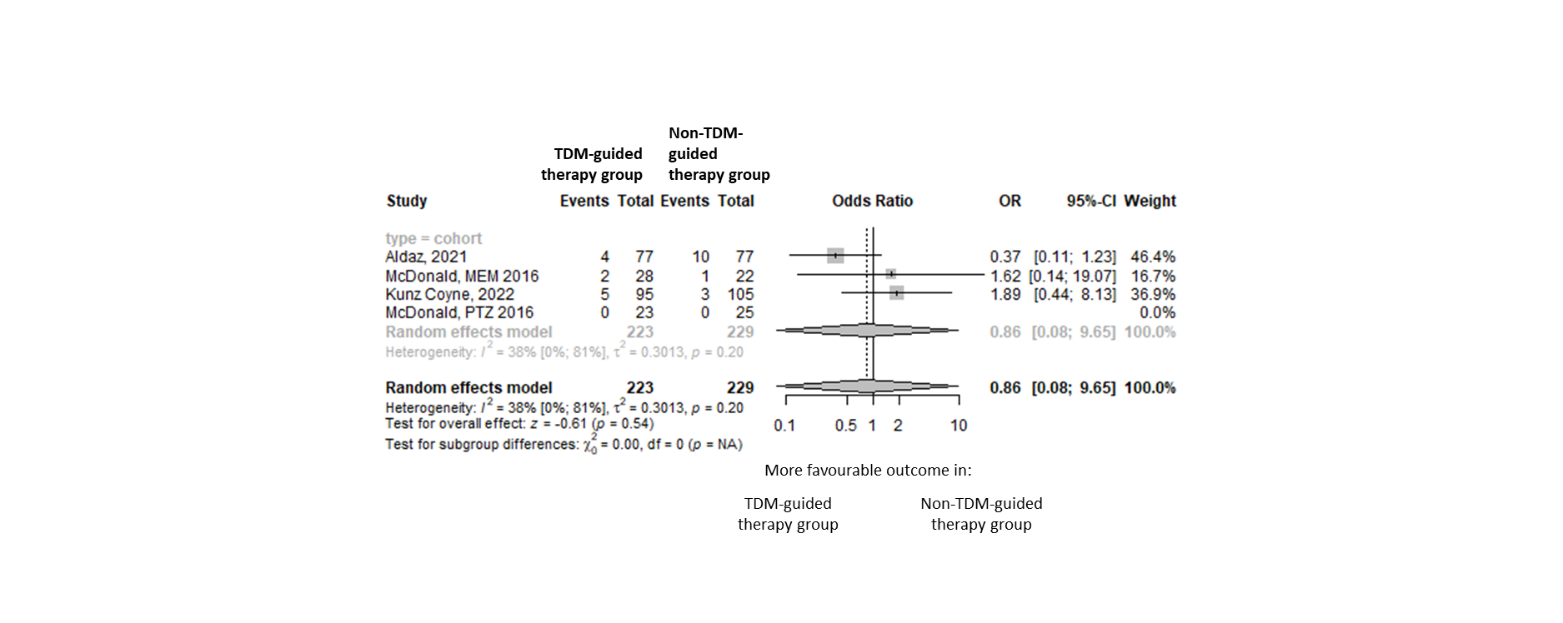
**

**S6. GRADE**

**Author(s):**

**Question:** TDM compared to Standard of care therapy for critically ill patients receiving beta-lactams

**Setting:**

**Bibliography:**

| **Certainty assessment** | | | | | | | **№ of patients** | | **Effect** | | **Certainty** | **Importance** |
| --- | --- | --- | --- | --- | --- | --- | --- | --- | --- | --- | --- | --- |
| **№ of studies** | **Study design** | **Risk of bias** | **Inconsistency** | **Indirectness** | **Imprecision** | **Other considerations** | **TDM** | **Standard of care therapy** | **Relative (95% CI)** | **Absolute (95% CI)** |  |  |
| **Clinical cure** | | | | | | | | | | | | |
| 3 | randomised trials | not serious | serious^a^ | not serious | not serious | none | 73/116 (62.9%) | 58/120 (48.3%) | **OR 1.91** (0.23 to 15.82) | **158 more per 1 000** (from 306 fewer to 453 more) | ⨁⨁⨁◯ Moderate | CRITICAL |
| **Microbiological eradication** | | | | | | | | | | | | |
| 2 | randomised trials | not serious | not serious | not serious | not serious | none | 47/69 (68.1%) | 38/70 (54.3%) | **OR 2.15** (0.00 to 6749.50) | **176 more per 1 000** (from -- to 457 more) | ⨁⨁⨁⨁ High | CRITICAL |
| **Clinical cure Obs** | | | | | | | | | | | | |
| 3 | observational studies | serious^b^ | not serious | not serious | not serious | none | 179/220 (81.4%) | 158/227 (69.6%) | **OR 1.98** (1.49 to 2.64) | **123 more per 1 000** (from 77 more to 162 more) | ⨁⨁⨁◯ Moderate | CRITICAL |
| **Microbiological eradication** | | | | | | | | | | | | |
| 2 | observational studies | not serious | not serious | not serious | not serious | none | 107/125 (85.6%) | 93/122 (76.2%) | **OR 1.89** (0.70 to 5.10) | **96 more per 1 000** (from 70 fewer to 180 more) | ⨁⨁⨁⨁ High | CRITICAL |
| **Treatment failure** | | | | | | | | | | | | |
| 3 | randomised trials | not serious | serious^a^ | not serious | not serious | none | 43/116 (37.1%) | 62/120 (51.7%) | **RR 0.52** (0.06 to 4.34) | **248 fewer per 1 000** (from 486 fewer to 1 000 more) | ⨁⨁⨁◯ Moderate | CRITICAL |
| **Treatment failure** | | | | | | | | | | | | |
| 2 | observational studies | not serious | not serious | not serious | not serious | none | 19/143 (13.3%) | 33/150 (22.0%) | **RR 0.55** (0.33 to 0.92) | **99 fewer per 1 000** (from 147 fewer to 18 fewer) | ⨁⨁◯◯ Low | CRITICAL |
| **Target attainment** | | | | | | | | | | | | |
| 3 | observational studies | not serious | not serious | not serious | not serious | none | 45/70 (64.3%) | 33/66 (50.0%) | **RR 1.84** (0.34 to 9.98) | **420 more per 1 000** (from 330 fewer to 1 000 more) | ⨁⨁◯◯ Low | CRITICAL |

**CI:** confidence interval; **MD:** mean difference; **OR:** odds ratio; **RR:** risk ratio

#### Explanations

a. Fournier 2018 reported opposite results

b. definition of clinical cure was heterogenous

**S7. Publications exlcuded from evaluation in the „Full text selection” phase**

| **Author, Year** | **Level of medical care** | **Population** | **Intervention** | **Control** | **Type of study** | **Antibiotic** | **Reason** | **DOI** |
| --- | --- | --- | --- | --- | --- | --- | --- | --- |
| Aardema et al, 2020 | tertiary referral hospital Groningen, the Netherlands | critically ill patients | continuous  infusion | intermittent infusion | randomized controlled trial | cefotaxime | did not fulfill inclusion criteria: intervention group did not receive TDM-adjusted dosages | 10.1093/jac/dkz463 |
| Abdul-Aziz et al, 2016 | ICUs of the  Tengku Ampuan Afzan Hospital, Kuantan and the  University Malaya Medical Centre, Kuala Lumpur, Malaysia | critically ill patients with severe sepsis | continuous infusion | intermittent infusion | randomized controlled trial | beta-lactam | did not fulfill inclusion criteria: intervention group did not receive TDM-adjusted dosages | 10.1007/s00134-015-4188-0\r10.1007/s00134-015-4188-0. |
| Abdulla et al, 2020 | ICUs of the Erasmus University Medical Center and Maasstad Hospital, Rotterdam, the Netherlands | critically ill patients | no intervention | measurement of antibiotic concentration | prospective observational study | beta-lactam | did not fulfill inclusion criteria:  there were no intervention and control groups | 10.1186/s13054-020-03272-z\r10.1186/s13054-020-03272-z. |
| Alshaer et al, 2022 | not defined | burn intensive care unit patients | ’selective’ TDM (performed for some patients) | ’universal’ TDM (performed for all patients) | retrospective chart review | beta-lactam | did not fulfill inclusion criteria: both patient groups underwent TDM-guided dosage adjustments | 10.1093/jbcr/irac099\r10.1093/jbcr/irac099. |
| Binder et al, 2013 | the hematology ward or the inten-  sive care station at the George August University Hospital,  Gottingen, Germany | intensive care unit patients | no intervention | measurement of standard meropenem dosings | prospective observational study | meropenem | did not fulfill inclusion criteria:  there were no intervention and control group | 10.1097/FTD.0b013e31827d496c\r10.1097/FTD.0b013e31827d496c. |
| Chiriac et al, 2021 | Heidenheim, Germany | critically ill patients with coronavirus disease 2019 (COVID-19) and pneumonia | no intervention | no standard of care group | retrospective single-center analysis | beta-lactam | did not fulfill inclusion criteria:  no intervention and control groups could be defined | 10.1097/md.0000000000026253\r10.1097/MD.0000000000026253. |
| Chiriac et al, 2021 | a German academic teaching hospital,  Germany | critically ill patients with sepsis or septic shock | no intervention | no standard of care group | retrospective study | piperacillin | did not fulfill inclusion criteria:  no intervention and control groups could be defined | 10.3390/antibiotics10060667\r10.3390/antibiotics10060667. |
| De Waele et al, 2012 | not defined | Conference abstract. | | | | | no full text was available | 10.1007/s00134-012-2683-0 |
| Drks, 2016a | not defined | No full text was found in the full-text selection. | | | | | no full text was available | <https://trialsearch.who.int/Trial2.aspx?TrialID=DRKS00011159> |
| Drks, 2016b | not defined | No full text was found in the full-text selection. | | | | | no full text was available | <https://trialsearch.who.int/Trial2.aspx?TrialID=DRKS00011023> |
| Euctr, E. S., 2021 | not defined | No full text was found in the full-text selection. | | | | | no full text was available | <https://trialsearch.who.int/Trial2.aspx?TrialID=EUCTR2019-004947-65-ES> |
| Euctr, N. L., 2018 | not defined | No full text was found in the full-text selection. | | | | | no full text was available | <https://trialsearch.who.int/Trial2.aspx?TrialID=EUCTR2017-004677-14-NL> |
| Hassanpour et al, 2021 | medical-surgical ICU of Imam Hossein Medical Center, affiliated with Shahid Beheshti University of Medical Sciences in Tehran, Iran | surgical intensive care unit patients with acute kidney disease | adjusted dose of meropenem | standard dose of meropenem | randomized controlled trial | meropenem | did not fulfill inclusion criteria:  non-eligible population (acute kidney disease) | 10.1007/s00228-020-03062-0\r10.1007/s00228-020-03062-0. |
| Lertwattanachai et al, 2020 | an academic hospital in  Bangkok, Thailand | patients with sepsis and septic shock | no TDM | standard dose | prospective randomized open-label study | meropenem | did not fulfill inclusion criteria:  no intervention was made | 10.1186/s40560-020-00442-7 |
| Machado et al, 2017 | tertiary care teaching Burn Intensive Care Unit of the Hospital das Clínicas, University of Sao Paulo, Brasil | patients at intensive care unit without age limit | antibiotic doses adjusted to plasma monitoring and pharmacokinetic modeling | conventional antimicrobial dose regimens | retrospective observational study | vancomycin, imipenem, meropenem, piperacillin | did not fulfill inclusion criteria:  pediatric patients and vancomycin therapy were included | 10.1016/j.clinthera.2017.06.008\r10.1016/j.clinthera.2017.06.008. |
| Mitton et al, 2021 | ICU at the Steve Biko Academic Hospital Pretoria, South Africa | critically ill surgical patients receiving imipenem | no intervention | imipenem trough levels measurement | retrospective observational study | imipenem | did not fulfill inclusion criteria:  no intervention was made | 10.7196/SAMJ.2021.v111i9.15634 |
| Mo et al,  2021 | Brooklyn Hospital Center,  community teaching hospital Brooklyn, New  York, United States of America | adult hospitalized patients with HAP or VAP | dose adjustment of cefepime | no dose adjustment of cefepime | retrospective observational study | cefepime | did not fulfill inclusion criteria:  patients were not critically ill | 10.1002/jcph.1967 10.1002/jcph.1967. |
| Moser et al, 2021 | tertiary care hospital, Bern, Switzerland | critically and non-critically ill patients | no intervention | measurement of flucloxacillin concentrations | prospective cohort study | flucloxacillin | did not fulfill inclusion criteria:  non-eligible population (non-critically ill included) | 10.1093/jac/dkab089\r10.1093/jac/dkab089. |
| Muller et al, 2018 | not defined | Conference abstract. | | | | | no full text available:  conference abstract | 10.1515/labmed-2018-0115 |
| Nct, 2018 | not defined | No full text was found in the full-text selection. | | | | | no full text available | <https://clinicaltrials.gov/show/NCT03645668> |
| Novy et al, 2020 | not defined | Letter to the editor. | | | | | no original data were provided: letter to the editor | 10.1016/j.accpm.2020.04.005\r10.1016/j.accpm.2020.04.005. Epub 2020 Apr 18. |
| Patel et al, 2012 | The Royal Brisbane and Women’s Hospital tertiary  referral burns center Queensland, Australia | burn patients | no intervention | no standard of care group | prospective observational study | beta-lactam | did not fulfill inclusion criteria:  no intervention was made | 10.1097/FTD.0b013e31824981a6\r10.1097/FTD.0b013e31824981a6. |
| Richter et al, 2019 | interdisciplinary German ICU of an academic teaching hospital, Germany | critically ill patients | continuous | no standard of care group | retrospective analysis | beta-lactam | did not fulfill inclusion criteria:  intervention group did not receive TDM adjusted dosages | 10.1007/s15010-019-01352-z\r10.1007/s15010-019-01352-z. |
| Roberts et al, 2010 | The Intensive Care Unit at Royal Brisbane and Women’s Hospital Brisbane, Australia | critically ill patients | no intervention | TDM | prospective observational study | beta-lactam | did not fulfill inclusion criteria:  no intervention was made | 10.1016/j.ijantimicag.2010.06.008\r10.1016/j.ijantimicag.2010.06.008. |
| Scharf et al, 2020 | two anaesthesiologically  managed ICUs of the university hospital in Munich, Germany | critically ill patients treated with meropenem or piperacillin | no intervention | three groups based on target attainment | prospective observational study | meropenem, piperacillin | did not fulfill inclusion criteria:  no intervention was made | 10.1186/s40560-020-00504-w\r10.1186/s40560-020-00504-w. |
| Sime et al,  2015 | The Queen Elizabeth Hospital in Adelaide,  Australia | febrile neutropenic patients with haematological malignancies | TDM with dose adjustment | TDM without dose adjustment | randomized controlled trial | piperacillin | did not fulfill inclusion criteria:  there was no evidence that patients had been treated at the ICU, or were critically ill | 10.1093/jac/dkv123\r10.1093/jac/dkv123. |
